# Cytidine diphosphate diacylglycerol synthase 2 is a synthetic lethal target in mesenchymal-like cancers

**DOI:** 10.1038/s41588-025-02221-2

**Published:** 2025-07-04

**Authors:** Tim Arnoldus, Alex van Vliet, Onno B. Bleijerveld, Adriaan F. H. de Groot, Qinglin Piao, Niek Blomberg, Désirée Schatton, Jing Dong, Susan E. van Hal-van Veen, Rolf Harkes, Anita E. Grootemaat, Natalie Proost, Birol Cabukusta, Christian Frezza, Marieke van de Ven, Nicole N. van der Wel, Martin Giera, Maarten Altelaar, Daniel S. Peeper

**Affiliations:** 1 Division of Molecular Oncology and Immunology, Oncode Institute, Netherlands Cancer Institute, Amsterdam, The Netherlands; 2 Proteomics facility, Netherlands Cancer Institute, Amsterdam, The Netherlands; 3 Center for Proteomics and Metabolomics, Leiden University Medical Center, Leiden, The Netherlands; 4 Cluster of Excellence Cellular Stress Responses in Aging-associated Diseases, University of Cologne, Faculty of Medicine and University Hospital Cologne, Institute for Metabolomics in Ageing, Cologne, Germany; 5 BioImaging Facility, Netherlands Cancer Institute, Amsterdam, The Netherlands; 6 Electron Microscopy Centre Amsterdam, Amsterdam University Medical Center, Amsterdam, The Netherlands; 7 Mouse Clinic for Cancer and Aging Preclinical Intervention facility, Netherlands Cancer Institute, Amsterdam, The Netherlands; 8 Department of Cell and Chemical Biology, Oncode Institute, Leiden University Medical Center, Leiden, The Netherlands; 9 Cluster of Excellence Cellular Stress Responses in Aging-associated Diseases, University of Cologne, Faculty of Mathematics and Natural Sciences, Institute of Genetics, Cologne, Germany; 10 Biomolecular Mass Spectrometry and Proteomics, Utrecht University, Utrecht, The Netherlands

**Keywords:** Data mining, Cancer therapy, Gene expression, Functional genomics, Chemical biology

## Abstract

Synthetic lethal interactions (SLIs) based on genomic alterations in cancer have been therapeutically explored. We investigated the SLI space as a function of differential RNA expression in cancer and normal tissue. Computational analyses of functional genomic and gene expression resources uncovered a cancer-specific SLI between the paralogs cytidine diphosphate diacylglycerol synthase 1 (*CDS1*) and *CDS2*. The essentiality of *CDS2* for cell survival is observed for mesenchymal-like cancers, which have low or absent *CDS1* expression and account for roughly half of all cancers. Mechanistically, the *CDS1–2* SLI is accompanied by disruption of lipid homeostasis, including accumulation of cholesterol esters and triglycerides, and apoptosis. Genome-wide CRISPR–Cas9 knockout screens in *CDS1*-negative cancer cells identify no common escape mechanism of death caused by *CDS2* ablation, indicating the robustness of the SLI. Synthetic lethality is driven by CDS2 dosage and depends on catalytic activity. Thus, CDS2 may serve as a pharmacologically tractable target in mesenchymal-like cancers.

## Abstract (web summary)

The paralogs cytidine diphosphate diacylglycerol synthase 1 and 2 form a potentially targetable synthetic lethal relationship in mesenchymal-like cancers that involves disruption of lipid metabolism.

## Main

SLIs can provide a therapeutic index in cancer^1^, as exemplified by the dependency on poly(ADP-ribose) polymerase (PARP) of *BRCA*-deficient hereditary breast and ovarian cancer. Whereas healthy cells retain one functional germline *BRCA* copy, somatic loss of the second copy results in cancer. As *BRCA* deficiency results in loss of homology-based DNA repair^2^, it causes a strong dependency on PARP for genomic stability and, hence, survival. This SLI has been clinically exploited with specific PARP inhibitors^3–5^. This clinical success, and other examples, have spurred efforts to identify additional cancer-associated SLIs^6–32^. Similar to *BRCA*–*PARP*, data on genomic alterations specific to cancer are commonly used to find SLIs^2–4,6–19,31^, such as the dependency on *WRN* in microsatellite unstable cancers^6,7^.

In addition to genomic alterations, cancers also have distinct gene expression profiles relative to healthy tissue. Publicly available datasets provide extensive transcriptional information as well as genetic dependencies in cancer. For example, the cancer Dependency Map (DepMap) is a comprehensive research initiative providing matched CRISPR screening and gene expression data for 913 cell lines across 30 cancer lineages (2021Q2)^33,34^. In addition, the Cancer Genome Atlas (TCGA) provides genome-wide gene expression data for 9,264 tumor and 741 healthy samples^35^. Together, these datasets provide 250 million datapoints on gene expression and genetic dependencies in cancer and can be mined to predict SLIs. For example, gene expression data have been used to retrospectively predict patient responses to targeted therapy using computational synthetic lethality analyses^29^.

Here, we performed unbiased computational analyses to find cancer-specific SLIs, taking advantage of these public functional genomic and gene expression resources, particularly the Genotype-Tissue Expression (GTEx) project, which provides genome-wide gene expression data for 17,382 healthy tissue samples^36^. We scored all ~340 million gene pairs for synthetic lethality and cancer specificity. We focused on the strong SLI that we uncovered between *CDS1* and *CDS2*, investigating cancer specificity, robustness of the synthetic lethal relationship in vitro and in mice, potential escape mechanisms, mechanism of cell death and lipidomic and proteomic analysis.

## Results

### *CDS1*–*2* form a common cancer-associated SLI pair

**Fig. 1:**
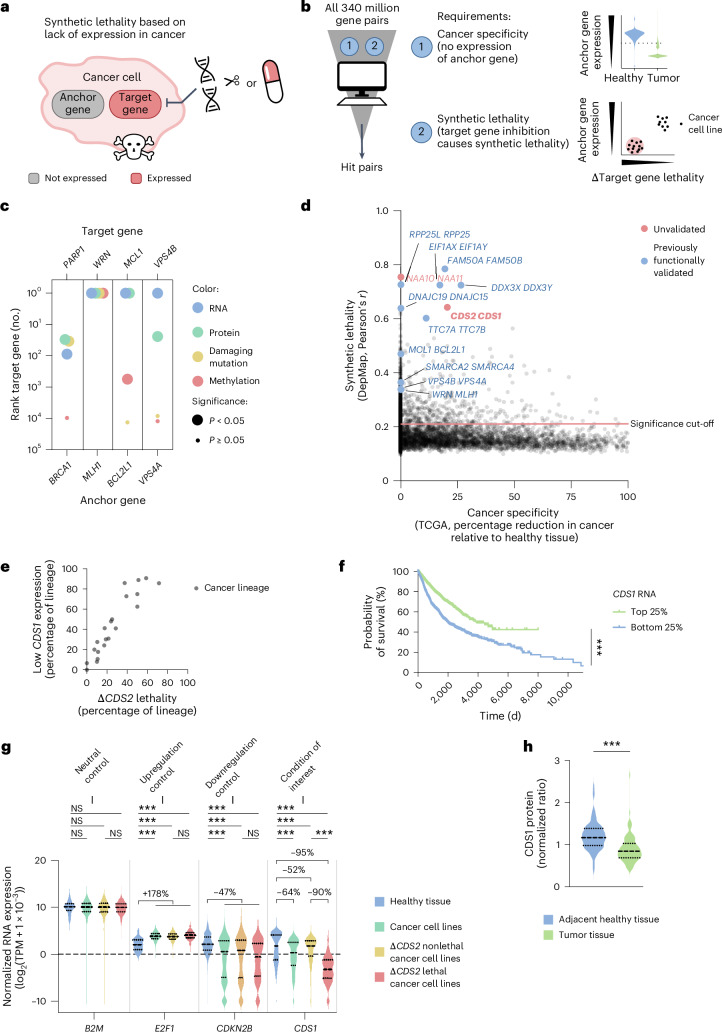
*CDS1*–*2* form a common cancer-associated SLI pair. **a**, Diagram depicting synthetic lethality based on lack of expression in cancer. **b**, Schematic depicting the computational analysis to identify cancer-specific SLIs. **c**, The correlation (Pearson’s *r*) of RNA, proteomic, mutation or methylation anchor gene data and gene dependency data in cancer cell lines for previously functionally validated synthetic lethal pairs. The rank of the known target gene partners was plotted for each data type. The significance cut-off was the top 5% enrichment (rank-based). *n* (cancer cell lines) = 913 (RNA), 299 (protein), 986 (damaging mutation) and 513 (methylation). **d**, Top predicted synthetic lethal pairs, named by their target gene and anchor gene, respectively. The *y* axis presents the correlation (Pearson’s *r*) between the anchor gene expression and target gene dependency (DepMap). The *x* axis presents the average reduction in anchor expression in patient tumor samples compared with patient healthy tissue samples (TCGA). Bonferroni-corrected *P* values from Pearson correlation tests were used to determine significance for synthetic lethality (*P* < 0.05). For the top synthetic lethal interactions, the significance of cancer specificity was assessed in Extended Data Fig. 1d,e. *n* = 913 cancer cell lines, 10,005 patient samples (9,264 tumor and 741 healthy). **e**, For each cancer lineage, the percentage of Δ*CDS2* lethal cancer cell lines plotted against the percentage of low *CDS1*-expressing cancer cell lines (DepMap). *n* = 906 cancer cell lines. **f**, Probability of survival for patients with the highest and lowest quartiles of *CDS1* expression in patient tumor samples (TCGA). The hazard ratio for the *CDS1*-low quartile was 1.90 [1.715–2.106] compared with the *CDS1*-high quartile. The log-rank Mantel–Cox test was used. *n* = 10,163 patients. **g**, Calibration gene-normalized RNA expression in healthy tissue samples (GTEx), cancer cell lines (DepMap), Δ*CDS2* lethal cancer cell lines (DepMap) and Δ*CDS2* nonlethal cancer cell lines (DepMap), presented in violin plots for the genes *B2M*, *E2F1*, *CDKN2B* and *CDS1*. The three dotted or dashed lines in each violin plot represent the quartile cut-offs. For significant differences, the percentage change in average TPM was indicated, with one overall average for controls. The Kruskal–Wallis test followed by Dunn’s test was used. *n* = 17,382 healthy tissue samples and 913 cancer cell lines (657 Δ*CDS2* nonlethal and 256 Δ*CDS2* lethal). **h**, Calibration gene-normalized CDS1 protein levels in matched healthy lung and lung tumor samples^47^. The three dotted or dashed lines in each violin plot represent the quartile cut-offs. The Mann–Whitney *U* test was used. *n* = 110 tumor samples and 102 adjacent healthy lung samples. In **f**, **g** and **h**: ***P < 0.001. NS, not significant. Source data

To query for SLIs based on differential gene expression between cancer and normal tissue, we set up a bioinformatic pipeline using public datasets on gene dependencies and RNA expression (Fig. 1a). This pipeline identifies anchor–target gene pairs, in which the anchor gene shows a relatively reduced expression level in cancer compared with normal tissue and in which disruption of the target gene results in cancer lethality (Fig. 1b).

**Extended Data Fig. 1:**
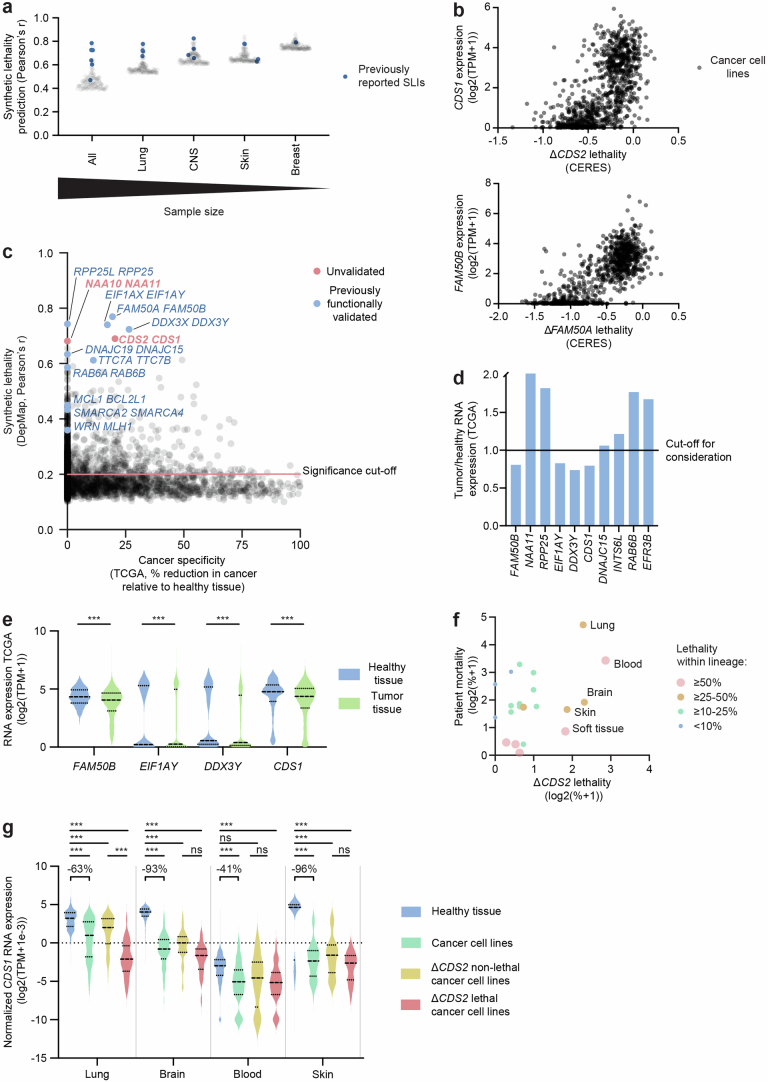
*CDS1-2* form a common cancer-associated SLI pair. **a**, Enrichment of previously functionally validated synthetic lethal interactions (*FAM50A-FAM50B*, *RPP25L-RPP25*, *EIF1AX-EIF1AY*, *DDX3X-DDX3Y*, *DNAJC19-DNAJC15*, *TTC7A-TTC7B, MCL1-BCL2L1*) for different sample sizes. The top 100 Pearson correlations between RNA and dependency data were plotted using all DepMap cancer cell lines or cancer type subsets. *n* (cancer cell lines) = 913 (all), 122 (lung), 70 (CNS), 63 (skin), 40 (breast). **b**, Top: plot depicting Δ*CDS2* lethality and *CDS1* RNA expression for cancer cell lines (DepMap). Bottom: plot depicting Δ*FAM50A* lethality and *FAM50B* RNA expression for cancer cell lines (DepMap). *n* = 913 cancer cell lines. **c**, Replication of the analysis and method of plotting presented in Fig. 1d which used 21Q2 CERES data using 23Q4 Chronos data (DepMap). *n* = 1,014 cancer cell lines, 10,005 patient samples (9,264 tumor). **d**, The average fold change in patient tumor samples compared to patient healthy samples (TCGA) for the anchor genes of the top ten predicted synthetic lethal pairs in Fig. 1d. *n* = 10,005 patient samples (9,264 tumor). **e**, The RNA expression in patient tumor and healthy samples for downregulated anchor genes in panel **d** (TCGA). Dotted lines represent the quartile cut-offs. Kruskal-Wallis test followed by Dunn’s test was used. *n* = 10,005 patient samples (9,264 tumor). **f**, For each cancer type the relative cancer mortality (SEER) was plotted against the frequency of Δ*CDS2* lethal cell lines (DepMap). The fraction of Δ*CDS2* lethal cell lines within each type is categorically depicted. *n* (cancer cell lines) = 906 (40 breast, 122 lung, 97 blood, 92 brain, 64 skin, 45 soft tissue). **g**, For the top lineages from panel **f**, the calibration gene-normalized *CDS1* expression in healthy tissue samples (GTEx), cancer cell lines (DepMap), Δ*CDS2* lethal cancer cell lines and Δ*CDS2* nonlethal cancer cell lines is presented. Dotted lines represent the quartile cut-offs. Kruskal-Wallis test followed by Dunn’s test was used. *n* (healthy) = 578 (lung), 2,642 (brain), 929 (blood), 1,809 (skin). *n* (cancer) = 122 (lung, 35 Δ*CDS2* lethal), 75 (brain, 25 Δ*CDS2* lethal), 48 (blood, 35 Δ*CDS2* lethal), 63 (skin, 24 Δ*CDS2* lethal). In **e** and **g**: ^***^P<0.001. ns, not significant. Source data

The DepMap includes additional types of data that we incorporated in our pipeline to score SLIs, including promoter methylation data, protein expression data, damaging mutation data and cancer-type data^33,34,37,38^. The pipeline uses combined gene dependency data from project SCORE (Sanger Institute) and project Achilles (Broad Institute). RNA expression data provided sufficient power to detect previously established SLIs, including *BRCA1*–*PARP1* and *WRN*–*MLH1* (Fig. 1c and Extended Data Fig. 1a)^3,4,6,7,10–13,25,26,39^, with *WRN*–*MLH1* serving as an example of the broader SLI between *WRN* and the microsatellite instability caused by *MLH1* deficiency. Although some SLIs may be best detected in single cancer types, we observed that analyzing all cancer types together improved resolution for identifying established interactions (Extended Data Fig. 1a). Therefore, we determined the SLI scores for all gene pairs with RNA expression data for all cancer types simultaneously.

The *CDS1*–*CDS2* and *NAA10*–*NAA11* gene pairs received high SLI scores (Fig. 1d, Extended Data Fig. 1b–e and Supplementary Table 1). However, the *NAA10*–*NAA11* SLI failed to show cancer specificity and was therefore not pursued. In contrast, the *CDS1*–*CDS2* SLI demonstrated significant cancer specificity. The high SLI score for *CDS1*–*CDS2* is consistent with other analyses and screens scoring *CDS1*–*CDS2* as a candidate synthetic lethal pair, but to our knowledge this has not yet been pursued^10,25,32,40^. CDS1 and CDS2 are enzymes that are conserved in plants and yeast. They serve to convert phosphatidic acid (PA) into cytidine diphosphate diacylglycerol (CDP-DAG), conceivably representing the bottleneck in phosphatidylinositol (PI) synthesis^41–43^. PI constitutes an essential component of cellular membranes and is also used as a critical kinase substrate regulating cell proliferation and survival^44,45^. Aberrations in PI signaling components act as common cancer drivers and are clinically targeted with phosphatidylinositol 3-kinase (PI3K) inhibitors^46^.

Our analysis confirmed *FAM50A*–*B*, *DDX3X*–*DDX3Y* and *EIF1AX*–*EIF1AY* as cancer-specific SLIs, in agreement with previous reports (Extended Data Fig. 1e)^10,25^. For several other SLIs, cancer specificity was previously inferred from genomic data^6,7,10,11,13^, but they did not score in the present study as such, for example, *WRN*–*MLH1*. This is conceivably the result of the relatively low frequency of genomic alterations in *MLH1* coupled to our focus on transcriptomic rather than genomic data.

In contrast, we noted that *CDS2* loss was associated with lethality in 19 out of 21 cancer types (Fig. 1e). Several of these concerned common cancers, including lung, blood, brain and skin cancers (Extended Data Fig. 1f). From here onwards, lethality as a function of *CDS2* loss will be referred to as Δ*CDS2* lethality. Patients with *CDS1*-low cancers showed significantly worse survival compared with patients with *CDS1*-high cancers (Fig. 1f). For these reasons we have focused here on the *CDS1*–*CDS2* SLI.

To validate and quantify cancer-specific loss of *CDS1* expression, we compared pan-cancer DepMap expression data and pan-tissue GTEx expression data (Fig. 1g). The data were calibrated on housekeeping genes, while control analyses on reference genes were performed to determine reliability. In line with the TCGA data (Fig. 1d), we observed common absence, or low expression, of *CDS1* in cancer cell lines compared with healthy tissue (64% average reduction, *P* < 0.0001). As expected, given the SLI, low or no *CDS1* expression strongly correlated with Δ*CDS2* lethality in cancer cell lines (90% average reduction, *P* < 0.0001). We also observed that lung, blood, brain and skin cancers have reduced *CDS1* levels compared with their lineages of origin (Extended Data Fig. 1g), suggesting that these cancers may suppress transcription of *CDS1* during cancer development. For lung cancer, differential CDS1 levels were confirmed using a cohort comprising patient-matched proteomics data from healthy and tumor tissue (Fig. 1h)^47^. Thus, our in silico analyses predict that the *CDS1–CDS2* gene pair constitutes a human cancer-associated SLI, specifically in common *CDS1*-low cancers.

### *CDS1* and *CDS2* are synthetic lethal across cancer types

**Fig. 2:**
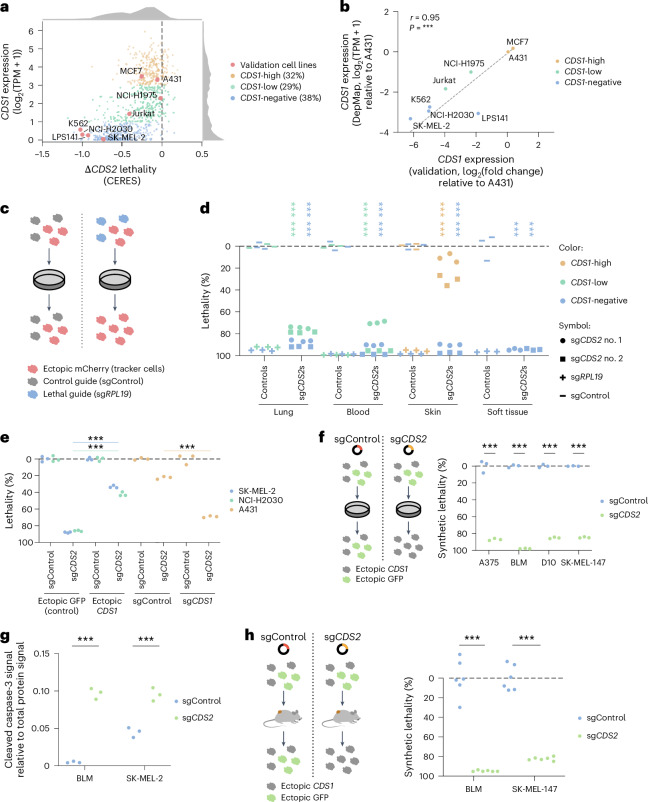
*CDS1* and *CDS2* are synthetic lethal across cancer types. **a**, Plot depicting synthetic lethality upon CRISPR perturbation of *CDS2* (Δ*CDS2* lethality) and *CDS1* RNA expression for cancer cell lines (DepMap). Cancer cell lines were categorized by *CDS1* RNA levels and cell lines used for validation are marked. The data distribution is depicted by a Δ*CDS2* lethality histogram (top) and a *CDS1* expression histogram (right). *n* = 913 cancer cell lines. **b**, Plot depicting *CDS1* RNA expression as measured by RNA sequencing (RNA-seq; DepMap) or qPCR analysis. The correlation (Pearson’s *r*) and associated *P* value were added. There was an average of two independent experiments with each *n* = 4 replicates for 8 cancer cell lines. **c**, Diagram depicting the method for quantifying lethality on CRISPR perturbation using fluorescent tracker cells. The mCherry-positive percentage was quantified by flow cytometry. **d**, Graph depicting cumulative lethality 14 d after CRISPR perturbation of *CDS2* using tracker cells (**c**). *CDS2* sgRNAs, positive control sgRNA (*RPL19*) and negative control sgRNA (sgControl) were included. Two-way ANOVA followed up by Dunnett’s test was used. *n* = 3 replicates for 7 cancer cell lines with 2 *CDS2* sgRNAs; see the 8-d measurement in Extended Data Fig. 2b. There is an independent experiment repeat in **e** (three cancer cell lines) and Fig. 3b (four cancer cell lines); the latter also includes an additional *CDS1*-high cancer cell line. **e**, For two *CDS1*-negative cell lines (NCI-H2030 and SK-MEL-2), *CDS1* or GFP (control) was ectopically expressed and the 14-d Δ*CDS2* lethality was determined using tracker cells (**c**). For one *CDS1*-high cell line *CDS1* (sg*CDS1* versus sgControl) was perturbed by CRISPR and the 8-d Δ*CDS2* lethality was determined using tracker cells (**c**). Two-way ANOVA followed by Tukey’s test was used. *n* = 3 replicates for 3 cancer cell lines. A431 and NCI-H2030/SK-MEL-2 are separate experiments. **f**, Left: diagram depicting method for quantifying synthetic lethality using ectopic *CDS1* or GFP expression. The GFP-positive percentage was quantified by flow cytometry. Right: graph depicting quantification of the 14-d synthetic lethality in melanoma. Two-way ANOVA followed by Šidák’s test was used. *n* = 3 replicates for 4 cancer cell lines; see the 7-d measurement in Extended Data Fig. 2d. **g**, Graph depicting snapshot of the total protein-normalized level of cleaved caspase-3 in Δ*CDS2* or control samples of BLM and SK-MEL-2 cell lines, as determined by quantitative western blotting. Two-way ANOVA followed by Šidák’s test was used. *n* = 3 replicates from independent lentiviral transductions for 2 cancer cell lines. **h**, Left: diagram depicting the in vivo variant of the method for quantifying synthetic lethality. Right: graph depicting quantification of the in vivo synthetic lethality in two melanoma cell-line models 17 d after subcutaneous melanoma tumor inoculation. Tumors were analyzed by flow cytometry. Two-way ANOVA followed by Šidák’s test was used. *n* = 6 NOD-scid *Il2rγ*-null mice per group each for 2 cancer cell lines. In **b** and **d**–**h**: ***P < 0.001. Source data

For wet lab validation of the computational predictions, we used a panel of cancer cell lines, which are also in the DepMap (Fig. 2a). Their *CDS1* RNA levels were confirmed by quantitative (q)PCR analysis (Fig. 2b). Low-throughput CRISPR perturbations were used to quantify the lethality inferred from genome-wide CRISPR knockout screens. By including fluorescent tracker cells serving as an internal control for each experimental condition, we quantified lethality over extended timeframes, showing both minimal variation and the effect size over time (Fig. 2c).

**Extended Data Fig. 2:**
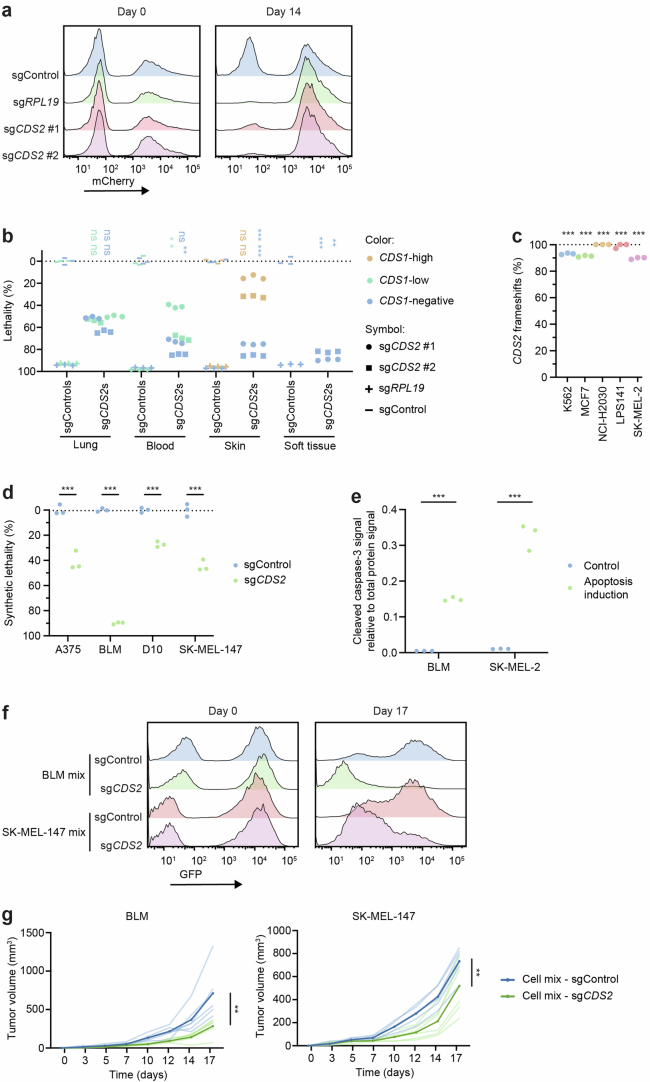
*CDS1* and *CDS2* are synthetic lethal across cancer types. **a**, As an example, representative histograms for the flow cytometry data used to calculate lethality for NCI-H2030 in Fig. 2d are presented. **b**, The 8-d results for the experiment in Fig. 2d. Two-way ANOVA followed up by Dunnett’s test was used. *n* = 3 replicates for 7 cancer cell lines with 2 *CDS2* sgRNAs. **c**, The percentage of cancer cells with frameshift upon sg*CDS2* compared to sgControl determined using TIDE. *n* = 3 replicates for 5 cancer cell lines. **d**, The 7-d results for the experiment in Fig. 2f. Two-way ANOVA followed by Šidák’s test was used. *n* = 3 replicates for 4 cancer cell lines. **e**, Graph depicting a snapshot of the total protein normalized level of cleaved caspase-3 in apoptotic or control samples of BLM and SK-MEL-2 cell lines, as determined by quantitative western blotting (Abby). Apoptosis was induced by TPCA-1 + TNF for BLM cells and staurosporine for SK-MEL-2 cells. Two-way ANOVA followed by Šidák’s test was used. *n* = 3 replicates for 2 cancer cell lines. **f**, Representative histograms for the flow cytometry data used to calculate synthetic lethality in Fig. 2h. **g**, Growth curves for the experiment in Fig. 2h. Note that the tumor growth curves are derived from cell mixes including both GFP-expressing and ectopically *CDS1*-restored cells. Two-way ANOVA was used. Mean is presented by dark lines. *n* = 6 NOD-scid *Il2rγ*-null mice per group for 2 cancer cell lines. In **b**-**e** and **g**: ^*^P<0.05, ^**^P<0.01, ^***^P<0.001. Source data

On perturbation of *CDS2* with one of two separate single guide (sg)RNAs, we were able to confirm Δ*CDS2* lethality in *CDS1*-low or *CDS1*-negative cell lines across several cancer types (Fig. 2d and Extended Data Fig. 2a–c). Furthermore, as predicted, a *CDS1*-high cell line was Δ*CDS2* nonlethal. Compared with the DepMap data, we observed strong lethality. Furthermore, Δ*CDS2* lethality was still evident in cell lines with low levels of *CDS1* RNA (Fig. 2d, green).

To validate the *CDS1* dependency of the Δ*CDS2* lethality, we either ectopically expressed *CDS1* in *CDS1*-negative cancer cell lines or perturbed *CDS1* in *CDS1*-proficient cancer cells. When *CDS1* was introduced into two *CDS1*-negative cell lines, the Δ*CDS2* lethality was largely negated. Conversely, when *CDS1* was perturbed in a *CDS1*-high cell line, it exhibited increased Δ*CDS2* lethality (Fig. 2e). To validate these observations, we admixed control green fluorescent protein (GFP)-expressing cancer cell lines with *CDS1*-restored cell lines in an additional panel of four human melanoma models. The results confirmed strong synthetic lethality on perturbation of *CDS2* in this panel (Fig. 2f and Extended Data Fig. 2d). Together, these functional experiments confirm that *CDS1* and *CDS2* constitute a synthetic lethal pair across a panel of cancer cell lines in vitro.

### Δ*CDS2* lethality is associated with apoptosis

We hypothesized that Δ*CDS2* lethality may result in apoptotic cell death in vitro. To investigate this, we collected samples from BLM and SK-MEL-2 cancer cell lines and measured cleaved caspase-3 by quantitative western blotting as a measure of apoptosis (Fig. 2g). For quantification, the cleaved caspase-3 signal was compared with the total protein signal in the same capillary. As positive controls, we analyzed cleaved caspase-3 levels on induction of apoptosis by TPCA-1 + tumor necrosis factor (TNF) for BLM cells and staurosporine for SK-MEL-2 cells (Extended Data Fig. 2e). Although it may be possible that, in some settings, other types of cell death may additionally occur, we observed a significant increase in cleaved caspase-3 in Δ*CDS2* cancer cells, indicating that Δ*CDS2* lethality is associated with tumor cell apoptosis.

### Δ*CDS2* lethality in vivo

Next, we investigated whether the SLI between *CDS1* and *CDS2* observed in silico and in vitro can be recapitulated in vivo. For this purpose, we again admixed control GFP-expressing cancer cell lines with *CDS1*-restored cell lines. Tumor cells were inoculated into immunodeficient NOD-scid *Il2rγ*-null mice and analyzed by flow cytometry of the tumors 17 d later. We observed a striking inability of *CDS2*-perturbed cells to contribute to tumor formation in vivo (Fig. 2h and Extended Data Fig. 2f,g; note that the tumor growth curves are derived from cell mixes including both GFP-expressing and ectopically *CDS1*-restored cells). Together, these results demonstrate that *CDS1* and *CDS2* form a robust synthetic lethal pair in cancer, in silico, in vitro and in vivo.

### No common escape mechanism for Δ*CDS2* lethality

**Fig. 3:**
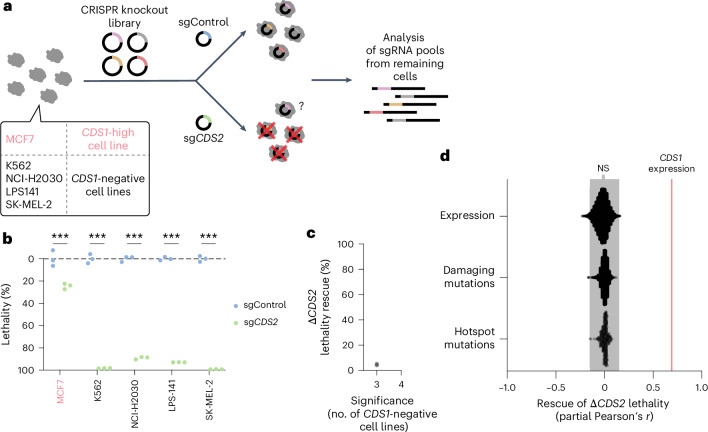
No common escape mechanism for Δ*CDS2* lethality. **a**, Diagram depicting the method used to screen for Δ*CDS2* lethality escape mechanisms. One *CDS1*-high cancer cell line and four *CDS1*-negative cancer cell lines were screened. **b**, The lethality of the screen cells between 15 d and 25 d. Lethality was quantified using fluorescent tracker cells as depicted in Fig. 2c. Two-way ANOVA followed by Šídák’s test was used. *n* = 3 replicates for 5 cancer cell lines. **c**, Graph depicting the screen hits identified in three of four or all four *CDS1*-negative cancer cell lines. The effect size was calculated using the average Δ*CDS2* lethality in the lethality assays with screen cells (**b**) and the fold-change enrichment of the second-best guides for each gene. Negative binomial modeling per sgRNA, followed by alpha-Robust Rank Aggregation per gene and Benjamini–Hochberg correction for the screens, was used to determine significance (*P* < 0.05). *n* = 1 for 4 cancer cell lines at 200× coverage with 4 sgRNAs per gene and 1,000 sgControls in the CRISPR library. **d**, Graph depicting Pearson’s *r* between Δ*CDS2* lethality and other features of cancer cell lines corrected for *CDS1* expression (DepMap). The correlation between Δ*CDS2* lethality and *CDS1* expression was added (red line). The features included were RNA expression, damaging mutations and hotspot mutations. Bonferroni-corrected *P* values from Pearson correlation tests were used. The gray box indicates the nonsignificant features. *n* (cancer cell lines) = 1,021. In **b**: ***P < 0.001. Source data

**Extended Data Fig. 3:**
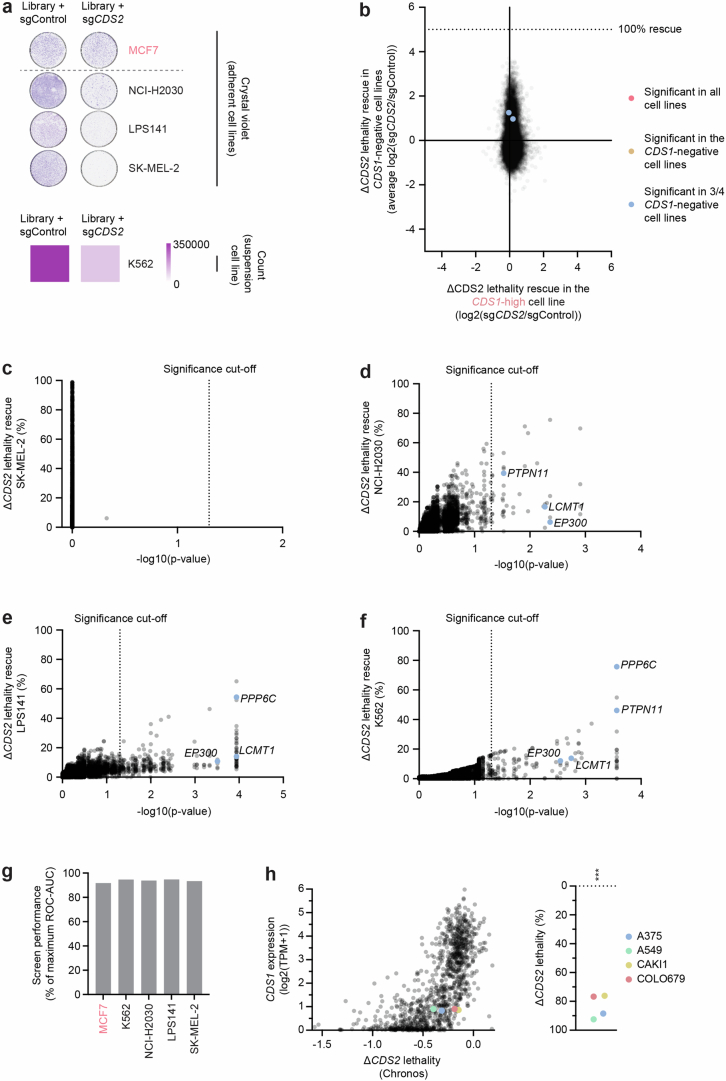
No common escape mechanism for Δ*CDS2* lethality. **a**, Top: Colony formation assays with adherent cell lines used for the screen (crystal violets). Bottom: Cell survival assay with the suspension screen cell line (cell counts). *n* = 1 for 5 cancer cell lines, an independent experiment was performed for quantification (Fig. 3b). **b**, The average Δ*CDS2* changes for knockouts of each other gene were plotted for the four *CDS1*-negative cancer cell lines against the *CDS1*-high cell line. The dotted line corresponds to the Δ*CDS2* change required to compensate for the average lethality quantified in Fig. 3b. *n* = 1 for 5 cancer cell lines at 200x coverage with 4 sgRNA per gene and 1,000 sgControls in the CRISPR library. **c-f**, Lethality rescue plotted against the Benjamini-Hochberg corrected *P* values from MAGeCK for each gene in the separate screens. Each of these panels presents the results for a different cancer cell line. The rescue was calculated using average lethality quantified in Fig. 3b. The two genes significant in three cell lines (*LCMT1* & *EP300*) and the two significant genes in two cell lines with the strongest rescue (*PPP6C* & *PTPN11*) were marked. Note that outliers above 100% were not plotted. *n* = 1 for 4 cancer cell lines at 200x coverage with 4 sgRNA per gene and 1,000 sgControls in the CRISPR library. **g**, As a quality control, each control arm was compared with the original library to detect essential genes (true positives) over non-essential genes (true negatives). The resulting area under the receiver operator curve (ROC-AUC) values for each screen are presented. A ROC-AUC above 90% of maximum is considered outstanding for CRISPR screens^86^. *n* = 1 for 5 cancer cell lines at 200x coverage with 4 sgRNA per gene and 1,000 sgControls in the CRISPR library. **h**, Left: Plot depicting Δ*CDS2* lethality and *CDS1* RNA expression for cancer cell lines (DepMap). Cancer cell lines used for validation are marked. Right: The 14-d lethality of the marked cancer cell lines. Lethality was quantified using fluorescent tracker cells as depicted in Fig. 2c. Two-way ANOVA followed by Šidák’s test was used. *n* = 1,014 cancer cell lines (DepMap, left) or 4 replicates for 4 cancer cell lines (right). In **h**: ^***^P<0.001. Source data

The computational and in vitro and in vivo functional validation data above demonstrate the broad cancer range and reproducibility of the *CDS1*–*2* SLI, prompting us to further challenge its robustness. Specifically, the rate-limiting role of the CDS enzymes in PI synthesis led us to investigate whether any cells can rewire their signaling network such that they can escape from this SLI. To investigate this in an unbiased, genome-wide fashion, we performed CRISPR knockout rescue screens in a panel of four *CDS1*-negative human cancer cell lines and, as a control, one *CDS1*-high cancer cell line (Fig. 3a and Supplementary Table 2). In parallel, cells from the screens were used to track Δ*CDS2* lethality during the screen. These analyses confirmed Δ*CDS2* lethality in four *CDS1*-negative cancer cell lines and extended our data on the lack of Δ*CDS2* lethality in *CDS1*-high cancer cell lines to an additional cancer cell line (Fig. 3b). In addition, colony formation assays were performed to visualize the lethal effect during the screen (Extended Data Fig. 3a; note that for K562 a different readout was used, because it is a suspension cell line).

Potential escape mechanisms were determined using Δ*CDS2* lethality quantified with tracker cells. However, the results of the screens (Fig. 3c and Extended Data Fig. 3b) indicated no common escape mechanism for Δ*CDS2* lethality. For example, the screen performed in SK-MEL-2 cells yielded no significant enrichment even after an additional 14 d (32 d in total; Extended Data Fig. 3c), while the other screens yielded only some cell-line-specific rescue (Extended Data Fig. 3d–f). Analysis of the dropout of essential genes confirmed the high quality of the screens (Extended Data Fig. 3g). To comprehensively cover all altered gene expression and DNA mutations, we also analyzed the DepMap for potential escape mechanisms (Fig. 3d). The results confirm that only *CDS1* expression commonly affects Δ*CDS2* lethality. We expanded our Δ*CDS2* lethality validation to also cover outliers in the DepMap data (Extended Data Fig. 3h): even for these we observed strong Δ*CDS2* lethality in cultured cancer cells. Although we cannot fully exclude the existence of specific escape mechanisms, together these findings suggest that, at least under the conditions tested in the present study, there are no common escape mechanisms for the combined loss of *CDS1* and *CDS2*, which is in agreement with the idea that CDS1 and CDS2 together serve as a bottleneck for PI synthesis.

### Mesenchymal-like cancers depend on *CDS2* for PI synthesis

**Fig. 4:**
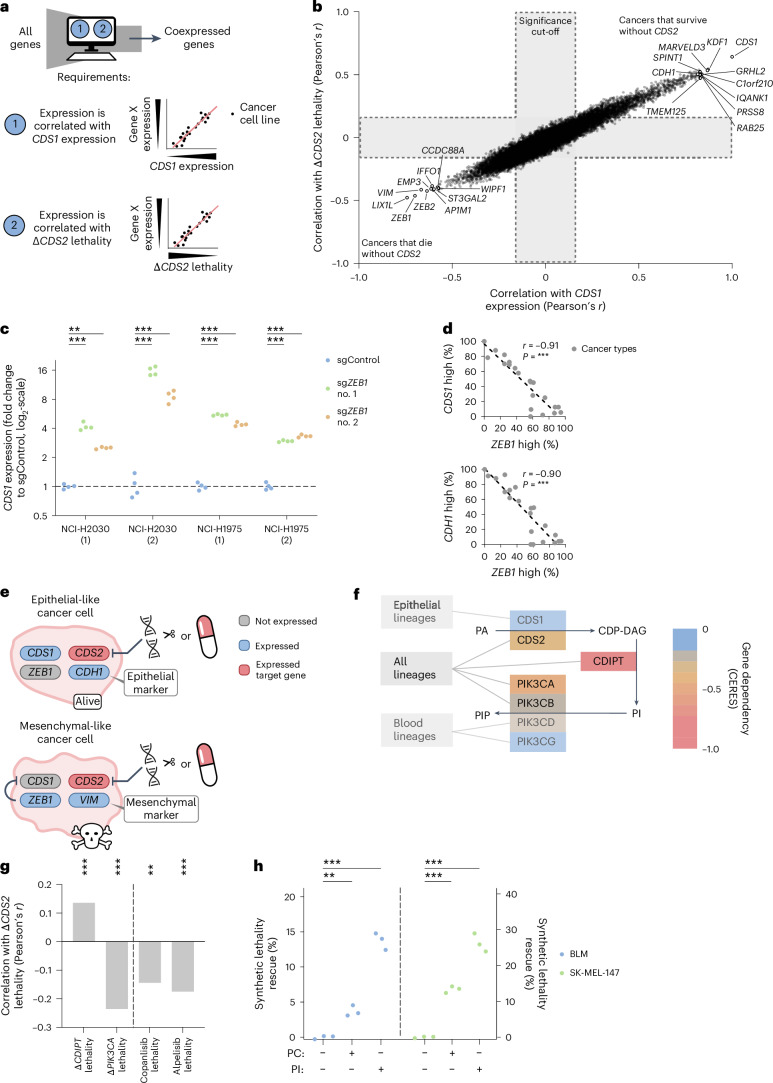
Mesenchymal-like cancers depend on *CDS2* for PI synthesis. **a**, Method for phenotyping cancer cell lines that are Δ*CDS2* lethal and *CDS1*-low compared to those that are Δ*CDS2* nonlethal and *CDS1*-high (DepMap) (illustrative). **b**, Genome-wide calculated Pearson correlations using the method depicted in **a** (DepMap). Representative genes enriched on either side are marked. Bonferroni-corrected *P* values from Pearson correlation tests were used to determine the significance cut-off. *n* = 913 cancer cell lines. **c**, Plot depicting *CDS1* RNA expression on *ZEB1* CRISPR perturbation as measured by qPCR analysis. The dashed line indicates the baseline of no change (1-fold change). Two-way ANOVA followed by Dunnett’s test was used for each cancer cell line. *n* = 4 replicates for 2 cancer cell lines in 2 independent experiments with each 2 sg*ZEB1* and 1 sgControl. **d**, For each cancer lineage, the percentage of *ZEB1*-high cancer cell lines (DepMap) was plotted against the percentage of *CDS1-*high or *CDH1*-high cancer cell lines. A linear regression, the associated Pearson correlation and associated *P* values were added. *n* = 906 cancer cell lines. **e**, Diagram depicting the characteristics of cancer cell lines with and without the *CDS1–CDS2* SLI. *CDH1* and *VIM* are representative epithelial and mesenchymal markers, respectively. **f**, Diagram depicting the core CDS pathway. The lethality on perturbation (color) and lineage-specific expression patterns (gray boxes on the left) of each enzyme in the pathway are indicated (DepMap). Gene dependency is represented by CERES. *n* = 913 cancer cell lines. **g**, Correlation between Δ*CDS2* lethality and Δ*CDIPT* lethality, Δ*PIK3CA* lethality, PIK3CA inhibitor lethality (alpelisib) or the pan-PI3K inhibitor lethality (copanlisib) in solid cancer cell lines (DepMap). Bonferroni-corrected *P* values from Pearson correlation tests were used. *n* (cancer cell lines) = 865 (*CDIPT* and *PIK3CA*), 428 (copanlisib) and 441 (alpelisib). **h**, Rescue of synthetic lethality on supplementation with PI or phosphatidylcholine (PC, control). The method to quantify synthetic lethality was introduced in Fig. 2f. PI and PC were complexed with lipid-free bovine serum albumin (BSA). Treatment was with 0.25 mM lipid for 7 d, started 7 d after transduction. Two-way ANOVA followed by Dunnett’s test was used. *n* = 3 replicates for 2 cancer cell lines. In **c**, **d**, **g** and **h**: ***P < 0.01, ***P < 0.001. Source data

**Extended Data Fig. 4:**
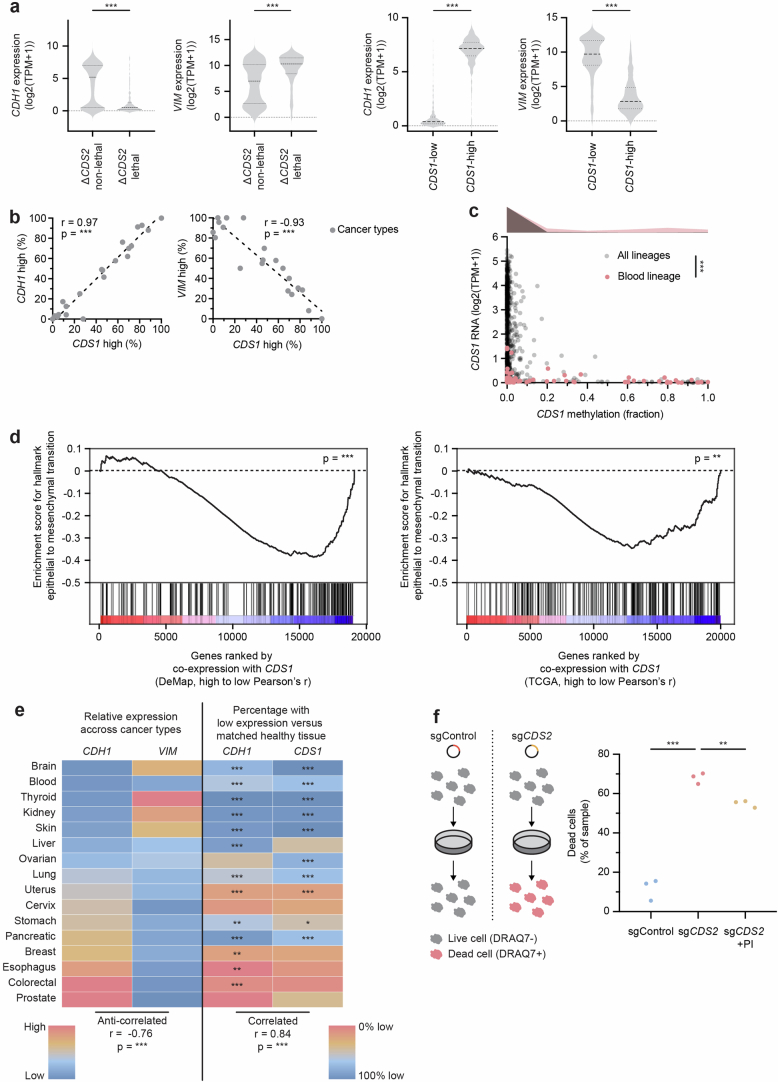
Mesenchymal-like cancers depend on *CDS2* for PI synthesis. **a**, *CDH1* and *VIM* expression in Δ*CDS2* lethal, Δ*CDS2* nonlethal, *CDS1*-low (bottom quartile) and *CDS1*-high (top quartile) cancer cell lines (DepMap). Mann-Whitney *U* test was used. *n* = 913 cancer cell lines (657 nonlethal, 256 lethal, 345 *CDS1* low, 352 *CDS1* high). **b**, The percentage of *CDS1*-high cancer cell lines (DepMap) was plotted against the percentage of *VIM-*high or *CDH1*-high cancer cell lines by type. A linear regression and Pearson correlation test were added. *n* = 906 cancer cell lines. **c**, *CDS1* promoter methylation in all cancer cell lines and the blood subtype (DepMap). Histograms show the distribution of *CDS1* promoter methylation (top). Mann-Whitney *U* test was used. *n* = 792 cancer cell lines (72 blood). **d**, The gene set enrichment analysis (GSEA) results for Hallmark epithelial to mesenchymal transition. Results are from a GSEA on genome-wide *CDS1* coexpression (Pearson’s r) using DepMap and TCGA data. The Benjamini-Hochberg corrected GSEA *P* values were used. *n* = 913 cancer cell lines, 11,069 patient samples. **e**, For 16 cancer types the calibration gene-normalized RNA expression in healthy tissue samples (GTEx) and cancer cell lines (DepMap) was compared. The cut-off for low expression of *CDS1* or *CDH1* expression was the bottom-tertile boundary for expression in healthy tissue samples of the same type (right). *CDH1* and *VIM* expression were plotted as a percentage of the highest expressing cancer type included (left). Kruskal-Wallis test followed by Dunn’s test was used by type. The Pearson correlation test was used across types. *n* (healthy/cancer) = 2,642/70 (brain), 929/48 (blood), 653/8 (thyroid), 89/25 (kidney), 1,809/63 (skin), 226/22 (liver), 180/53 (ovarian), 578/122 (lung), 142/32 (uterus), 19/13 (cervix), 359/29 (stomach), 328/42 (pancreatic), 459/40 (breast), 1,445/28 (esophagus), 966/50 (colorectal), 245/5 (prostate). **f**, Left: the method for quantifying the dead cell fraction using the DNA-intercalating dye DRAQ7. Right: rescue of Δ*CDS2* cell death upon overnight supplementation with 2 mM phosphatidylinositol (PI) started 11 d after transduction of BLM cells. One-way ANOVA followed by Šidák’s test was used. *n* = 3 replicates for 1 cancer cell line. In **a**-**f**: ^*^P<0.05, ^**^P<0.01, ^***^P<0.001. Source data

To understand which cancer types show reduced *CDS1* expression and, hence, Δ*CDS2* lethality, we characterized *CDS1*-low cancers using publicly available data (Fig. 4a). First, we noted that *CDS1*-high cancer cells express high levels of the epithelial marker gene E-cadherin (*CDH1*), whereas *CDS1*-low cancer cell lines express mesenchymal markers like *ZEB1*, *ZEB2* and vimentin (*VIM*) (Fig. 4b and Extended Data Fig. 4a,b)^48,49^. Mechanistically, this is in agreement with the notion that *CDS1* expression is suppressed in mesenchymal cancers by the transcription factor ZEB1, previously reported to bind the *CDS1* locus and suppress its expression^50,51^. Therefore, we measured the effect of sgRNAs for *ZEB1* on *CDS1* expression by qPCR and computationally scored the correlation between the expression of *ZEB1* and *CDS1* or *CDH1* across cancer types in the DepMap. Both results support the notion that *ZEB1* suppresses *CDS1* expression in mesenchymal-like cancers (Fig. 4c,d). Besides this major regulatory mechanism, we also observed that a rare subset of the blood lineage cancer cell lines exhibits methylation of the *CDS1* promoter (Extended Data Fig. 4c). *CDS1* methylation did not correlate with *ZEB1* expression. Overall, these findings indicate that the suppression of *CDS1* expression in mesenchymal-like cancers by *ZEB1* results in their strong dependency on *CDS2* (Fig. 4e).

Gene set enrichment analysis (GSEA) indicated that *CDS1*-low cancers are enriched for the Hallmark epithelial–mesenchymal transition (EMT) gene set (*P* = 0.0001 DepMap, *P* = 0.009 TCGA; Extended Data Fig. 4d). Mesenchymal-transitioned cancers are common, and often highly metastatic and therapy-refractory^48,52–55^. These findings are in agreement with our observations that low *CDS1* expression is common, more frequent in cancers compared with their healthy tissue of origin and associated with worse survival (Fig. 1e,f and Extended Data Fig. 1g). Furthermore, when expanding this comparison with healthy tissue to 16 cancer types covering *CDS1* and *CDH1*, we observed that both genes are expressed at low levels in mesenchymal-like cancer cell lines (Extended Data Fig. 4e). Together, these results suggest that suppression of *CDS1* expression is an integral element of EMT in cancer.

Next, we zoomed in on the pathway in which CDS1 and CDS2 are involved, as has been defined by previous studies (Fig. 4f)^41–43,56^. We incorporated expression and dependency data from the DepMap for the enzymes involved. In line with the requirement for CDS1 or CDS2, the next enzyme in the pathway, cytidine diphosphate diacylglycerol synthase inositol-3-phosphatidyltransferase (CDIPT), is essential for survival. In addition, *CDS2* and *CDIPT* are significantly co-dependent in DepMap cancer cell lines, which is indicative of a similar mechanism of dependency (Fig. 4g)^28,57^. Accordingly, when supplementing the cell culture medium with exogenous PI, we observed a significant rescue of cell death, suggesting that Δ*CDS2* lethality is due, at least in part, to insufficient availability of PI (Fig. 4h and Extended Data Fig. 4f).

CDS2 acts upstream of the PI3K signaling module, regulating growth and survival. Unexpectedly, DepMap analysis revealed that Δ*CDS2* lethality is not accompanied by lethality with either genetic loss of *PIK3CA* or pharmacological PIK3CA inhibition (alpelisib). Instead, we observed a strong anticorrelation between Δ*CDS2* lethality and Δ*PIK3CA* lethality (Fig. 4g). This anticorrelation was independent of PI3K isoforms, as judged by pan-PI3K inhibitor responses. These results demonstrate an essential role of *CDS* for PI synthesis in mesenchymal cancers. Furthermore, they unexpectedly point to a contribution of *CDS1* and *CDS2* to survival signaling independent of the classic PI3K pathway.

### Cholesterol production is induced on *CDS1+CDS2* ablation

**Fig. 5:**
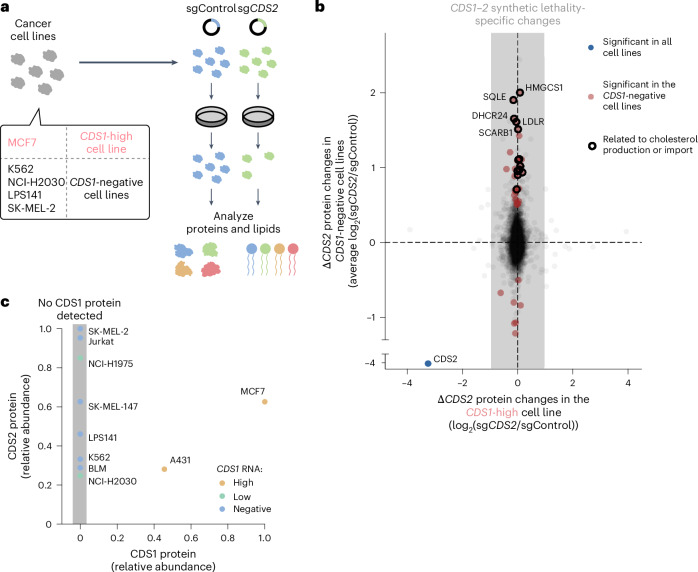
Cholesterol production is induced on *CDS1+CDS2* ablation. **a**, Diagram depicting the samples generated for quantifying proteins and lipids (Fig. 6). One *CDS1*-high cancer cell line and four *CDS1*-negative cancer cell lines were analyzed. **b**, Average Δ*CDS2* protein changes for the four *CDS1*-negative cancer cell lines (*y* axis) plotted against the Δ*CDS2* protein changes in the *CDS1*-high cancer cell line (*x* axis). The significance was determined using the Student’s *t*-test. *n* = 4 replicates for 5 cancer cell lines. An independent experiment repeat using a second independent instrument is shown in Extended Data Fig. 5c for SK-MEL-2. **c**, CDS1 protein levels plotted against CDS2 protein levels for a panel of ten cancer cell lines. *CDS1* expression from public RNA-seq was added as in Fig. 2b (DepMap) with additional data for BLM and SK-MEL-147 (accession no. SRP132830). For each axis, the relative abundance to the highest cancer cell line included was plotted. Data were normalized to the median levels of calibration proteins and the mean of the experiments was used. CDS1 was detected in all MCF7 and A431 replicates, but no other replicates. *n* = 3 and 4 replicates from 2 independent experiments. Source data

To biochemically define the effects of *CDS2* perturbation in *CDS1*-negative cancer cells, we performed multiomic analyses in a panel of four *CDS1*-negative cancer cell lines and, as a control, one *CDS1*-high cancer cell line (Fig. 5a). Proteomics was performed to identify deregulated cellular processes on CDS2 loss. In addition, lipidomics was performed to quantify buildup or depletion of lipids, specifically those in the CDS pathway.

**Extended Data Fig. 5:**
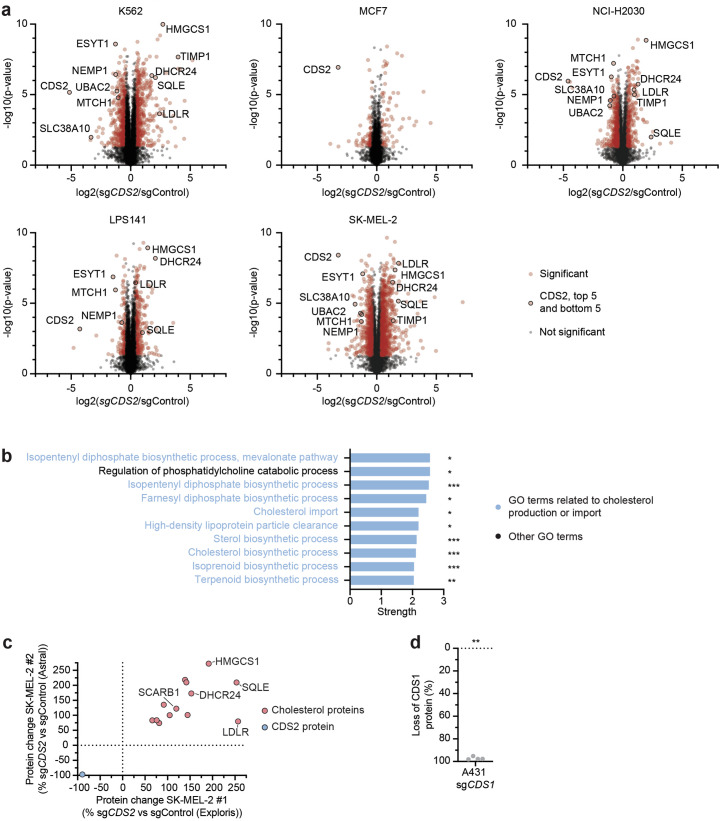
Cholesterol production is induced upon *CDS1+CDS2* ablation. **a**, Volcano plots presenting the Δ*CDS2*-induced protein changes in five cancer cell lines. In addition to CDS2, the top and bottom five proteins significant in at least three out of four *CDS1*-negative cancer cell lines were marked. *n* = 4 replicates for 5 cancer cell lines. **b**, The top ten significantly enriched GO terms among Δ*CDS2*-changed proteins in the four *CDS1*-negative cancer cell lines. Benjamini-Hochberg corrected *P* values from Stringdb were used. *n* = 4 replicates for 4 cancer cell lines. **c**, Graph depicting a second independent experiment for SK-MEL-2 using a different instrument (*y* axis, Astral). Data is plotted against the experiment presented in Fig. 5b (*x* axis). All plotted changes were significant in both experiments. Only the 13 cholesterol-related proteins from Fig. 5b and CDS2 were plotted. Significance was determined by Student’s t-test. *n* = 4 replicates for 1 cancer cell line in two independent experiments. **d**, For one *CDS1*-high cell line *CDS1* (sg*CDS1*, versus sgControl) was perturbed by CRISPR and the change in CDS1 protein level was plotted. Significance was determined by Student’s t-test. *n* = 4 replicates for 1 cancer cell line. In **b** and **d**: ^*^P<0.05, ^**^P<0.01, ^***^P<0.001. Source data

The proteomics analysis allowed for quantification of ~7,000 proteins in each cell line (Supplementary Table 3). As expected, the levels of CDS2 protein had dropped tenfold in all five cell lines on *CDS2* CRISPR perturbation (Fig. 5b and Extended Data Fig. 5a). In addition, large groups of proteins were commonly and significantly down- or upregulated in *CDS1*-low cancer cell lines, suggesting an orchestrated response (Fig. 5b and Extended Data Fig. 5a). Gene ontology (GO) term enrichment analysis of these proteins revealed major induction of cholesterol import and production on Δ*CDS2* lethality (Extended Data Fig. 5b), which was a robust effect across cancer cell lines and different experiments and proteomics analyses (Fig. 5b and Extended Data Fig. 5a,c). Overall, we observed that *CDS2* perturbation in *CDS1*-negative cancer cells results in upregulation of cholesterol proteins, including HMGCS1, SQLE, DHCR24, LDLR and SCARB1.

We expanded the proteomics to cover also CDS1 protein by using a different method (Methods). As expected, on CRISPR perturbation of *CDS1*, the level of CDS1 protein dropped by 97% (Extended Data Fig. 5d). We observed that CDS1 protein was clearly detected in all replicates in *CDS1* RNA high cancer cell lines. However, it was not detectable in the *CDS1* RNA-low or -negative cancer cell lines (log_2_(transcripts per kilobase per million mapped reads (TPM)) < 3) in a panel of 10 cancer cell lines (Fig. 5c).

### Lipid homeostasis requires either *CDS1* or *CDS2* expression

**Fig. 6:**
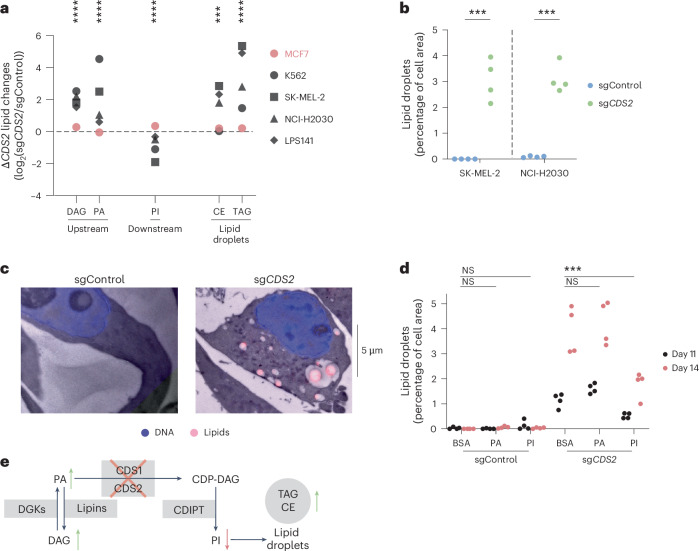
Lipid homeostasis requires either *CDS1* or *CDS2* expression. **a**, Five Δ*CDS2* lipid class changes presented (full results in Extended Data Fig. 6a). The asterisks indicate the number of *CDS1*-negative cell lines with at least *P* < 0.01 significant lipid alterations compared with the *CDS1*-high cell line. Two-way ANOVA followed by Šidák’s test was used. *n* = 4 replicates for 5 cancer cell lines. **b**, Cell areas staining positive for the lipid compartment dye BODIPY in live-cell imaging. Two-way ANOVA followed by Šidák’s test was used. *n* = 4 replicates for 2 cancer cell lines; see **d** for an independent experiment for one cancer cell line. **c**, Representative combined light and transmission electron microscopy image of an sgControl and an sg*CDS2* SK-MEL-2 cell stained for lipids (Nile Red) and DNA (Hoechst). *n* = 1 for 1 cancer cell line. Two independent light microscopy experiments were performed for quantification (**b** and **d**). **d**, Lipid droplet quantification on supplementation with 0.25 mM PI or PA in SK-MEL-2. PI and PA were complexed with lipid-free BSA. Treatment was started at 8 d and refreshed at 11 d. Two-way ANOVA followed by Šidák’s test was used. *n* = 4 replicates for 1 cancer cell line. See **b** an for independent experiment repeat without lipid treatments, also covering one additional cancer cell line. **e**, Diagram depicting lipid compartments and the CDS2 pathway. The Δ*CDS2* lipid class changes in the four *CDS1*-negative cell lines are marked by arrows. DGKs, diacylglycerol kinases. In **a** the significance was indicated by one asterisk for each cell line with *P* < 0.01. In **b** and **d**: ***P < 0.001. Source data

**Extended Data Fig. 6:**
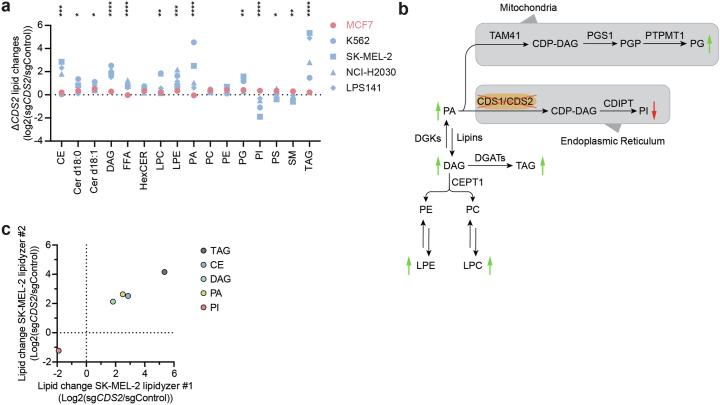
Lipid homeostasis requires either *CDS1* or *CDS2* expression. **a**, Δ*CDS2* lipid changes, presented in alphabetic order. Only lipid classes detected in all samples were plotted. Stars indicate number of *CDS1*-negative cancer cell lines with at least two-star significant lipid alterations compared to the *CDS1*-high cancer cell line. For abbreviations see bottom of figure description. Two-way ANOVA followed up by Šidák’s test was used. *n* = 4 replicates for 5 cancer cell lines. **b**, CDS2 pathway with Δ*CDS2*-changed lipid classes in *CDS1*-negative cancer cell lines marked with arrows. For abbreviations see bottom of figure description. Included enzymes are annotated on UniProt. **c**, Graphs depicting sg*CDS2* lipid changes in a second independent experiment for SK-MEL-2 (*y* axis). Key lipids from Fig. 6a were plotted. Data is plotted against the Fig. 6a data (*x* axis). All plotted changes were significant. Two-way ANOVA followed up by Šidák’s test was used. *n* = 4 replicates for 1 cancer cell line in 2 independent experiments. In **a**: Significance was indicated by one * for each cell line with *P*< 0.01. Lipids: CE = cholesterol esters, Cerd18:0 = ceramide d18:0, Cerd18:1 = ceramide d18:1, DAG = diacylglycerol, FFA = free fatty acids, HexCER = hexosylceramides, LPC = lysophosphatidylcholine, LPE = lysophosphatidylethanolamine, PA = phosphatidic acid, PC = phosphatidylcholine, PE = phosphatidylethanolamine, PG = phosphatidylglycerol, PI = phosphatidylinositol, PS = phosphatidylserine, SM = sphingomyelin, TAG = triglycerides. Enzymes: PEMT = phosphatidylethanolamine n-methyltransferase, CEPT1 = choline/ethanolamine phosphotransferase 1, DGATs = diacylglycerol o-acyltransferases, DGKs = diacylglycerol kinases, CDS = cytidine diphosphate diacylglycerol synthase, TAM41 = mitochondrial phosphatidate cytidylyltransferase, CDIPT = cytidine diphosphate diacylglycerol synthase inositol-3-phosphatidyltransferase, PGS1 = phosphatidylglycerophosphate synthase 1, PTPMT1 = protein tyrosine phosphatase mitochondrial 1. Source data

Lipidomic analysis allowed for quantification of the major lipid classes in the cells, with phosphorylated PI (PIP) serving as the major signaling molecule^44^ (Extended Data Fig. 6a and Supplementary Table 4). Major changes as a function of *CDS1* expression were robustly detected on CDS2 loss (Fig. 6a and Extended Data Fig. 6a–c; note that the stars indicate the number of *CDS1*-negative cancer cell lines with *P* < 0.01). For example, cholesterol esters (CEs) and triglycerides (TAGs) were massively upregulated. Furthermore, we observed strong buildup of CDS2 substrates and depletion of the downstream product PI.

In line with the buildup of CEs and TAGs, *CDS2*-perturbed cells formed large lipid droplets^58^, which were visible by both light and electron microscopy, staining positively for the lipid dyes BODIPY (boron dipyrromethene) and Nile Red. Quantification of BODIPY-stained live cells revealed that on average 3% of the imaged *CDS2*-knockout cells were made up of lipid (Fig. 6b). Combined light and electron microscopy of fixed cells showed these lipid droplets in more detail (Fig. 6c). Supplementation with the downstream CDS product PI significantly reduced the percentage area of lipid droplets in *CDS2*-perturbed cells (Fig. 6d). Combined with the lack of lipid droplets with the CDS substrate PA in control cells, this suggests that a shortage of PI drives the formation of most of the droplets. Together, these findings again corroborate that CDS signaling represents a biochemical bottleneck to support lipid homeostasis, demonstrating that lethality upon *CDS2* perturbation in *CDS1*-low cell lines is accompanied by major changes in lipid metabolism and induction of the cholesterol pathway (Fig. 6e).

### CDS2 dosage and catalytic activity govern synthetic lethality

**Fig. 7:**
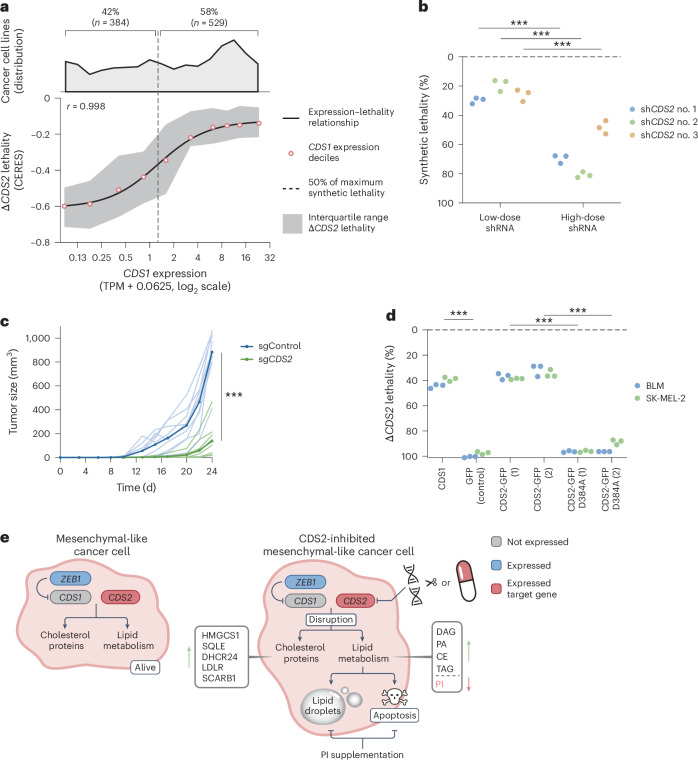
CDS2 dosage and catalytic activity govern synthetic lethality. **a**, The relationship between *CDS1* expression and Δ*CDS2* lethality. Deciles for cancer cell line *CDS1* expression were plotted against Δ*CDS2* lethality using DepMap data, with variation in Δ*CDS2* lethality indicated by the interquartile range. A sigmoid curve was fitted through the *CDS1* expression deciles with *r* = 0.998. Hill’s equation yielded a log_2_(TPM + 0.0625) of 1.273 as the level of *CDS1* expression, with 50% of maximum synthetic lethality among the cancer cell line collection (dashed line). For the totals above and below this level, numbers and percentages are shown on top, together with a histogram of the cancer cell line distribution. *n* = 913 cancer cell lines (DepMap). **b**, Graph depicting quantification of the 10-d synthetic lethality in BLM using the method for quantifying synthetic lethality with ectopic *CDS1* or GFP expression described in Fig. 2f. Two-way ANOVA followed by Šidák’s test was used. *n* = 3 replicates for 1 cancer cell line for 3 sh*CDS2*. **c**, Tumor growth after subcutaneous inoculation of control and *CDS2*-perturbed BLM melanoma cells. Two-way ANOVA was used (*n* = 9 NOD-scid *Il2rγ*-null mice per group for 1 cancer cell line). **d**, For two *CDS1*-negative melanoma cancer cell lines *CDS1*, *GFP* (control), *CDS2*–GFP or *CDS2*–GFP Asp384Ala was ectopically expressed and the 14-d Δ*CDS2* lethality was determined using tracker cells (Fig. 2c). Two-way ANOVA followed by Šidák’s test was used. *n* = 3 replicates for 2 cancer cell lines. The *CDS2*–GFP and *CDS2*–GFP Asp384Ala cell line variants were each generated in duplicate via independent transductions (no. 1, 2). Δ*CDS2* lethality in *CDS1* or *GFP* variants was replicated in one independent experiment for SK-MEL-2 (Fig. 2e) and two independent experiments for BLM (Fig. 2f: in vitro; Fig. 2h: in vivo). **e**, A diagram summarizing the mechanistic aspects of *CDS1–CDS2* synthetic lethality. On the left, upregulated cholesterol proteins are listed. On the right, buildup of the substrate lipids (PA, DAG) and of the lipid droplet lipids (CEs, TAGs) and depletion of the product PI is indicated. In **b**–**d**: ***P < 0.001. Source data

**Extended Data Fig. 7:**
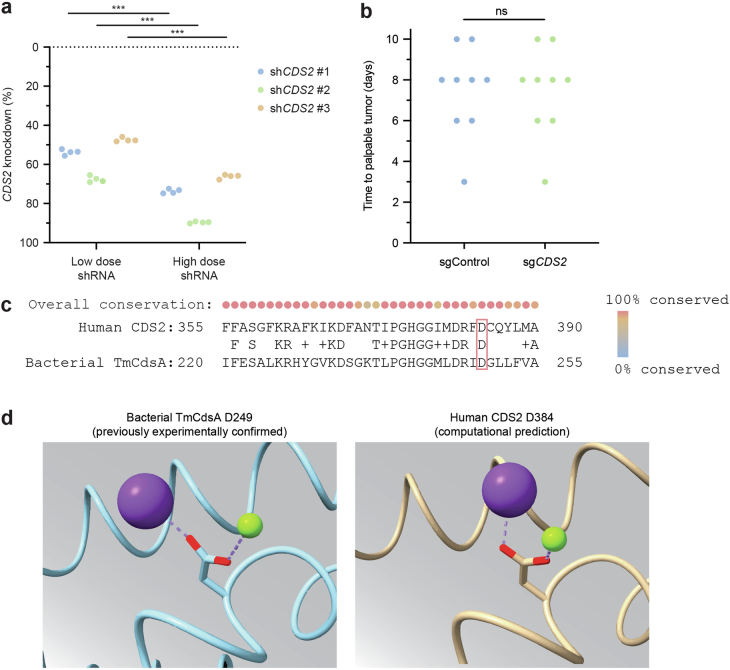
CDS2 dosage and catalytic activity govern synthetic lethality. **a**, Graph depicting RNA quantification by qPCR of 10-d *CDS2* knockdown in ectopic *CDS1*-restored BLM cells from the experiment depicted in Fig. 7b. Two-way ANOVA followed up by Šidák’s test was used. *n* = 3 replicates for 1 cancer cell line for 3 sh*CDS2*. **b**, Plot depicting time until palpable tumors were formed for the experiment in Fig. 7c. Student’s t-test was used. *n* = 9 NOD-scid *Il2rγ*-null mice per group. **c**, Alignment of a part of the human CDS2 amino acid sequence with bacterial TmCdsA showing they both contain an aspartic acid (red box). Added on top is the conservation across 150 species of each of the presented amino acids as determined by ConSurf^62^. **d**, The computationally predicted (Alphafill) ion binding to the aspartic acid highlighted in panel **c** for bacterial TmCdsA and human CDS2^63^. The bacterial structure was validated in a previous report. In **a**: ^***^P<0.001. Source data

Given that our in silico, in vitro and in vivo results demonstrate that *CDS1* and *CDS2* form a robust synthetic lethal pair in mesenchymal-like cancers, we set out to investigate any favorable characteristics for pharmacological inhibition. First, we investigated the dose dependency of *CDS1–CDS2* synthetic lethality. Using DepMap data, we observed that the relationship between *CDS1* expression and Δ*CDS2* lethality closely follows a dose–response-like relationship (Fig. 7a). Of cancer cell lines, 42% have at least half the maximum synthetic lethality. This is in line with our data linking low *CDS1* expression to mesenchymal-like cancers and the notion that roughly half of all cancers are mesenchymal like (Fig. 4a–d and Extended Data Fig. 4a,b,d,e)^59,60^. We used low and high doses of *CDS2* short hairpin (sh)RNA to generate two levels of *CDS2* knockdown (Extended Data Fig. 7a), which confirmed the dose-dependent lethality (Fig. 7b). Together, these data support a dose-dependent essentiality of CDS signaling for survival in cancer cell lines.

Next, we investigated whether the observed in vivo SLI between *CDS1* and *CDS2* can be recapitulated without admixing the cancer cells. For this purpose, tumor cells were inoculated into immunodeficient NOD-scid *Il2rγ*-null mice. We observed an identical time to establishment of palpable tumors in mice injected with control or C*DS2-*perturbed tumor cells (Extended Data Fig. 7b). However, robust Δ*CDS2* lethality manifested in time, again demonstrating the SLI in vivo, now with a homogeneous population of *CDS2*-ablated tumor cells (Fig. 7c).

In line with the idea that CDS1 and CDS2 form a biochemical bottleneck in lipid homeostasis, we hypothesized that loss of the catalytic activity of CDS2 drives Δ*CDS2* lethality. To study this, we designed a catalytically inactive variant of CDS2 based on TmCdsA from *Thermotoga* *maritima*, a thermophilic bacterium, for which this was previously defined (Extended Data Fig. 7c,d)^61–63^. As expected, add-back of either *CDS1* or catalytically proficient *CDS2* rescued Δ*CDS2* lethality compared with the control (Fig. 7d). However, Δ*CDS2* lethality could not be rescued by add-back of catalytically inactive *CDS2*.

## Discussion

Genomic alterations in cancer can serve as a mechanistic basis for SLIs^3,4,6–19,31^. Cancers also show distinct gene expression patterns compared with healthy tissue, prompting us to explore the cancer SLI space based on differential gene expression in cancer and healthy tissues. Taking advantage of extensive publicly available cancer datasets, we identified by computational mining that CDS2 serves as a synthetic lethal target in mesenchymal-like cancers. Our bioinformatic pipeline revealed a potential therapeutic index because of differential *CDS1* expression in cancer cells (low) and normal cells (high). We confirmed that *CDS1* and *CDS2* constitute a functional SLI across many cancer types, particularly mesenchymal-like cancers, representing roughly half of all cancers^59,60^, including both EMT cancers and cancers with a mesenchymal origin. The SLI is mechanistically explained by the fact that the combined loss of *CDS1* and *CDS2* is incompatible with cell survival, which, in combination with the low or absent expression of *CDS1* in mesenchymal-like cancer cells, results in Δ*CDS2* lethality (Fig. 7e). Our data are in agreement with results by Chan et al. (reported in the companion paper to this manuscript^64^) who reported that *CDS1* and *CDS2* are a synthetic lethal gene pair in uveal melanoma.

A series of CRISPR knockout rescue screens not only confirmed the synthetic lethal relationship between *CDS1* and *CDS2*, but also that they form a bottleneck in a pathway required for PI synthesis that apparently is essential for (cancer) cell viability. We found no common escape mechanism by CRISPR screening and computational analyses. However, ectopic *CDS1* introduction rescues Δ*CDS2* lethality and is compatible with cancer cell survival. Taken together, these observations suggest that it may not be straightforward for cancer cells to restore *CDS1* expression upon inactivation of one of its regulators to rescue cell death on *CDS2* inactivation. An example is the mesenchymal transcription factor ZEB1, depletion of which induces *CDS1*, but also reduces cancer cell survival^33,50,51^. Mechanistically, we show that Δ*CDS2* lethality is associated with cancer cell apoptosis, which can be rescued at least in part by supplementing exogenous PI. The latter is consistent with a requirement for CDS2 to produce PI. Our data also strongly suggest that, unexpectedly, PI synthesis is required for cell survival independent of classic PI3K-dependent survival signaling, because cell lines that depend on *CDS1* or *CDS2* for survival did not require PI3K. Our data imply that CDS2 inhibition may be explored to eliminate particularly mesenchymal-like cancers, which are common, highly metastatic and relatively resistant to therapy^48,52–55^.

To date, no inhibitor is available for CDS2 targeting. However, as our data demonstrate that its enzymatic activity is involved, pharmacological targeting may be possible. For example, for PI3K and diglyceride acyltransferase (DGAT), enzymes acting in the same pathway, inhibitors exist, while for PI3K inhibition, isoform-specific inhibitors are also available^46^. In addition, we observed that essentiality of CDS2 is dose-dependent, indicating that it may be amenable to pharmacological inhibition. As dependency data for healthy tissues are not included in the DepMap data, we cannot exclude that dose-limiting toxicities for CDS2 inhibition may occur. Proliferating fibroblasts are mesenchymal-like, but given that they are mostly involved in wound healing or are cancer-associated^65,66^, their targeting may not cause severe toxicity or even be clinically beneficial. Furthermore, the GTEx data suggest that high expression of *CDS1* is ubiquitous across healthy tissues while CDS2-targeting morpholinos are tolerated in mice^67^. We would propose developing a CDS2-specific inhibitor to maximize the therapeutic index; this may be possible given the different acyl-chain preferences for CDS2 and CDS1 (ref. ^43^). Clearly, a CDS2-specific inhibitor would be required to determine whether, as our large-scale computational and functional data in vitro and in vivo predict, a therapeutic index for clinically exploiting the *CDS1–2* SLI indeed exists.

## Methods

### Software

Flow cytometry data were collected using FACSDiva (v.8.0) and analyzed using Flowjo (v.10.6.0). For data analysis and statistics, Excel (v.16.71), R (v.4.2.2), RStudio (v.2023.03.0 + 386) or GraphPad Prism (v.9.4.1 or v.10.0.3) was used. The quantitative elements of figures were generated using GraphPad Prism or Flowjo. The visual elements of graphs and plots were designed and generated in Adobe Illustrator (v.26.2). MAGeCK v.0.5.9.5 was used for screen analysis^68^. For live-cell imaging and analysis, ZEN (v.3.5), FIJI (v.2.14.0), Java (v.1.8.0) and ImageJ (v.1.54f) were used^69^. ChimeraX v.1.5rc202210210730 was used for protein visualization^70^. ConSurf and AlphaFill were used for evaluating conservation^62,63^. DIA-NN (v.1.9 or v.1.9.1), Proteome Discoverer (Thermo Fisher Scientific, v.3.1.0.638) using Chimerys on Ardia (v.1.0.0-qf.1.) and Perseus (v.1.6.15.0) were used for proteomics analysis (Supplementary Note)^71,72^. For gene set enrichment, GSEA (v.4.1.0) or STRING was used. For combined light and electron microscopy analysis, LAS X (v.3.7.3.23245), MAPS (v.3.17) and ICY ec-CLEM-v2 (v.ICY-82440) were used. For genome-wide screen sequencing BCLConvert (v.3.9.2-3.e17), Perl (v.5.010) and xcalibr (v.0.12031) were used. For qPCR, Quantstudio (v.1.5.2) was used.

### Nomenclature

HGNC (HUGO Gene Nomenclature Committee) gene names and UniProt protein names were used.

### Cell line culture

Cell lines were cultured in Dulbecco’s modified Eagle’s medium (DMEM; Gibco, cat. no. 41966052) 10% fetal calf serum (FCS; Sigma-Aldrich, cat. no. 3101120) or Roswell Park Memorial Institute (RPMI; Thermo Fisher Scientific, cat. no. 21875034) 10% FCS for lung and blood cancer cell lines. Cells were passaged at subculturing ratios corresponding to 80% confluence at the next passage. During experiments, medium was supplemented with penicillin and streptomycin (Invitrogen, cat. no. 15140122).

Authenticated cell lines were used by STR profiling cell lines with a kit (Promega, cat. no. B9510) or delivering samples to Eurofins (Supplementary Table 5). Public references were available for all cell lines except SK-MEL-147 and BLM. For SK-MEL-147, a reference was requested via private correspondence^73^. *Mycoplasma*-negative cells were used by screening cells in culture for *Mycoplasma* spp. monthly^74^.

### Cancer-specific synthetic lethality prediction

Briefly, to rank potential synthetic lethal candidate pairs, Pearson’s correlation between fitness data (CERES or Chronos) and messenger RNA expression, proteomics, mutation or methylation data were calculated with 21Q2 or 23Q4 DepMap data^34,75^. We used Pearson’s *r* because it measures the strength and direction of the linear relationship between two variables, in this case anchor gene status and target gene dependency. For methylation and mutation, the ranking is reversed, because more mutation or methylation corresponds with less gene activity (whereas more RNA or protein corresponds with more gene activity). To validate this approach, several positive control, synthetic lethal pairs were selected. Positive control analyses were run using all pairs with the anchor genes.

To identify the most promising new SLIs, the anchored approach was replaced with a genome-wide analysis encompassing 340 million gene pairs. Each pair includes the anchor gene (expression gene) and the target gene (fitness effect gene), respectively. For each pair we calculated Pearson’s correlation between the expression of the anchor gene and Δtarget gene lethality across all cancer cell lines included. From the results, the top pair for each anchor was ranked to form a hit list. All duplicate target genes were removed to avoid false positives arising from anchor gene coexpression with other anchor genes. The change in expression was calculated for each anchor gene in the synthetic lethality prediction using reanalyzed TCGA healthy and tumor RNA data^76^. Increases in expression were plotted as a 0% reduction in Fig. 1d.

To analyze the DepMap for escape mechanisms, we used 23Q4 DepMap data and calculated the partial Pearson’s correlation between the expression of each gene and Δ*CDS2* lethality across all cancer cell lines corrected for *CDS1* expression. We did the same for damaging and hotspot mutation data, also correcting for *CDS1* expression. We corrected for *CDS1* expression to exclude false positives from *CDS1* expression coexpressed or co-mutated features.

### Cut-offs used in analyses

For Fig. 1e,g and Extended Data Fig. 1f,g, Δ*CDS2* lethal cancer cell lines were selected by CERES <−0.5. For context, a CERES of −1 corresponds to the core essential, whereas a CERES of 0 corresponds to the nonessential^33^. For Fig. 1e, low *CDS1*-expressing cancer cell lines were selected by log_2_(TPM + 1) < 1.5. For Fig. 4d and Extended Data Fig. 4b, the cut-offs for high expression were put right before the high peak on the histograms (log_2_(TPM + 1) of 2.5 for *CDS1*, 4.5 for *CDH1*, 2 for *ZEB1* and 6 for *VIM*).

### Patient mortality and survival analysis

The 21Q2 DepMap data for cell lines with available RNA and dependency data were used for Δ*CDS2* lethality. Lineages of patient mortality data and DepMap data were matched manually (Supplementary Table 6).

### Comparing CDS1 expression between healthy tissue and cancer cell lines

GTEx v.8 TPM was downloaded from the portal on 1 January 2022. The 21Q2 DepMap data were downloaded from the DepMap portal. Calibration genes with available TPM in DepMap and GTEx were used for normalization^77^. Up- and downregulation controls were selected from the literature^78,79^. Briefly, the TPM for each calibration gene was normalized to their own average expression. Then the TPM for *B2M*, *CDS1*, *CDKN2B* and *E2F1* was normalized to the average of the calibration gene expression. Calibrated results are similar to uncalibrated results as a result of intrinsic total RNA calibration of the GTEx and DepMap dataset TPM data. The analysis was also performed for blood, skin, lung and brain lineage data, with the original labeling from the DepMap and GTEx, also including soft-tissue atypical teratoid rhabdoid tumors, originally labeled ATRT, in the DepMap brain category. This was expanded to 16 cancer types using minor labeling changes for lineage matching between datasets.

### Healthy and tumor proteome data

Tumor and normal adjacent lung proteome data from Satpathy et al. were downloaded from entry PDC000234 and analyzed^47^. Specifically, log_2_-transformed ratios to internal controls were collected and sample names were mapped to their healthy or tumor tissue origin. Before plotting and statistical testing log_2_-transformation was undone, resulting in ratios to internal control. The internal control was reported to be a mix of all samples. Still, calibration genes with available ratios in all samples were used for normalization^77^. Each calibration gene ratio was normalized to its own average ratio. Then, the ratio for CDS1 was normalized to the geometric mean of the normalized calibration gene ratios for each sample.

### Quantitative PCR to quantify RNA

Three housekeeping genes for calibration were selected from a previously described list of options based on stable pan-cancer cell line expression in the DepMap^77^. The qPCR was performed as previously described^80^. The average of quadruplicate cycle threshold (*C*t) values was calculated for each gene. The −ΔΔ*C*t method was used for analysis: To normalize to calibration genes, the geometric mean of the three calibration gene *C*t values was subtracted from the *CDS1* *C*t value. To normalize to A431, the *C*t value of A431 was subtracted from the other cell line *C*t values. By base-2 exponential transformation and multiplication by −1, *C*t values were transformed to fold-changes. One representative calibration gene (*VPS29*) was selected and used for Fig. 4c and Extended Data Fig. 7a. Primer sequences are available in Supplementary Table 7.

### Lentiviral transduction

Cell lines were lentivirally transduced as previously described^80^. Transduced cells were always kept on selection during cell culture in vitro. The complete selection was verified by the loss of the control untransduced cells on selection. Low-throughput CRISPR perturbation using pLentiCRISPR v.2 Puro or Blast and ectopic expression using pLX304 Blast (*CDS1*, *CDS2*–*GFP*, *CDS2*–*GFP Asp384Ala* and GFP)^81^, pLKO.1 Puro or pCDH Puro (mCherry) was performed as previously described^82,80^. SHC002 (shControl) and TRCN00000s ending on 35859, 35861 and 35862 (sh*CDS2* nos. 1–3) from MISSION TRC-Hs 1.5 (human) were used. The shRNA virus was concentrated 40× (Millipore, cat. no. UFC910024). For high-dose transduction, it was used undiluted (sh*CDS2* nos. 2 and 3) or 1:10 diluted (sh*CDS2* no. 1) with a final protamine sulfate (Sigma-Aldrich, cat. no. 1101230005) concentration of 8 µg ml^−1^. For low-dose transductions, it was used 1:100 diluted without protamine sulfate. For Fig. 2e Blast resistance was introduced to mCherry-transduced cells using pLentiCRISPR v.2 Blast. In Fig. 2d, sg*CDS2* no. 1 and no. 2 were used and for all other experiments sg*CDS2* no. 2 was used. The sg*CDS1* was introduced using pLentiCRISPR v.2 Blast. The sgRNA sequences are available in Supplementary Table 7.

### Cloning *CDS2*–GFP and *CDS2*–GFP Asp384Ala

The pLX304 Blast containing *CDS2* was edited to generate *CDS2*–GFP and *CDS2*–GFP Asp384Ala^81^. Carboxy-terminal GFP was added with a flexible glycine and serine linker sequence (Supplementary Table 7). The sg*CDS2* no. 2 target site sequence was altered with silent mutations (Supplementary Table 7). For the mutant, a nonsilent sequence change was made to encode an Asp384Ala amino acid substitution (5′–3′: GAC → GCC). Cloning involved ligation with T4 DNA ligase, restriction with NheI + SbfI (GFP addition, Asp384Ala mutation) and restriction with XmaI + SbfI (silent mutations). Gene blocks were ordered from IDT and enzymes were from New England Biolabs (NEB). *CDS2*–GFP and *CDS2*–GFP Asp384Ala plasmids are available on request.

### Determining (synthetic) lethality by tracking fluorescent cells

Briefly, after lentiviral transduction (1 d) and puromycin selection of sgRNA-transduced cells (2 d), the indicated cell mixes were prepared. For the in vivo experiments cells were selected 1 d longer. To determine lethality, sgRNA-transduced cells were mixed with non-sgRNA-transduced, mCherry-positive cells. On the day of mixing (day 0) and at the indicated later time points, samples from each cell mix were analyzed by flow cytometry to determine the ratio between negative and positive cells. The final ratios were normalized to day 0 ratios and sgControl ratios. For lethality, normalized ratios were transformed into percentages to obtain survival percentages and subtracted from 100 to obtain lethality percentages. To determine synthetic lethality ectopic *CDS1*- or GFP-expressing cells, both transduced with the same sgRNA, were mixed on day 0. Then, lethality was calculated for the GFP-positive cells in an identical manner as for the mCherry-negative cells. For Extended Data Fig. 3h and Fig. 7d, unmixed mCherry-positive cells were used to determine the fraction of background mCherry-negative cells among the mCherry-positive cells. This fraction was multiplied with the mCherry-positive cell number from each mixed sample and the resulting expected background was subtracted from the respective mCherry-negative cell counts of that sample.

### Flow cytometry

**Extended Data Fig. 8:**
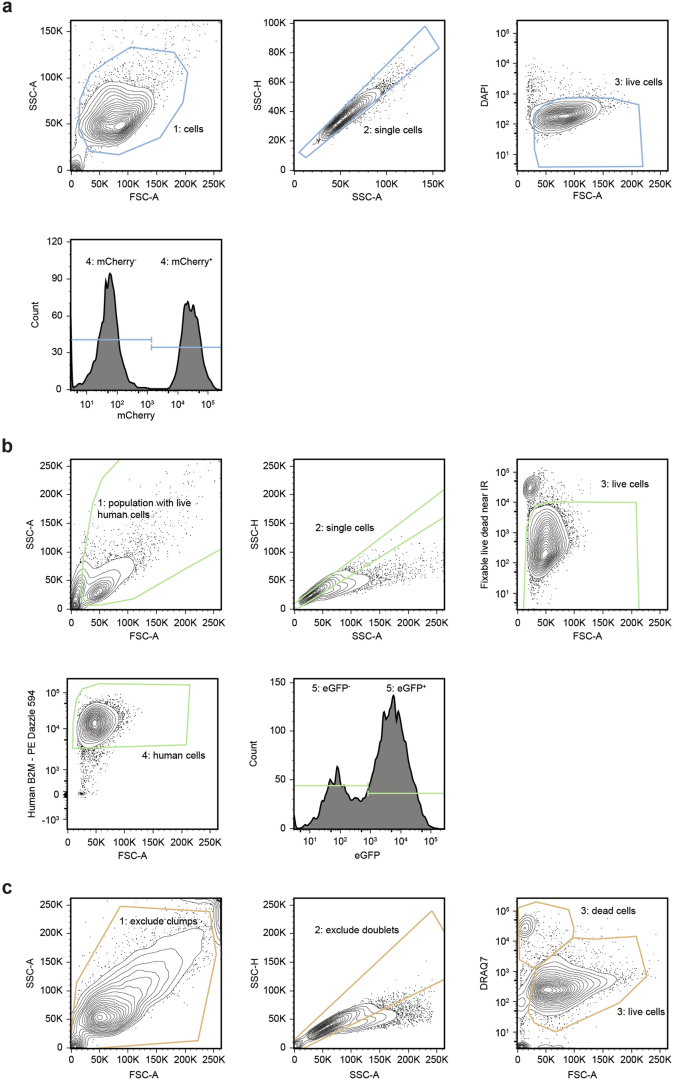
Gating strategies. **a**, The gating strategy for in vitro GFP or mCherry mixing experiments. **b**, The gating strategy for the in vivo mixing experiment. **c**, The gating strategy for dead cell quantification.

Flow cytometry data were collected on a BD Fortessa flow cytometer, regularly maintained in the Netherlands Cancer Institute (NKI) flow cytometry facility. One of several live or dead dyes was always included: DAPI (Sigma-Aldrich, cat. no. D9542-50MG), PI (Merck, cat. no. 537059-100MG), DRAQ7 (Thermo Fisher Scientific, cat. no. D15106) and fixable near infrared (Thermo Fisher Scientific, cat. no. L34976). BSA, 0.1%, in phosphate-buffered saline (PBS) was used as a washing and staining solution. Samples were washed 3× (including resuspension as one wash) in 0.1% BSA in PBS, once for dead cell experiments to preserve dead cells and 6× for in vivo samples to reduce postdigestion debris. The gating strategies used are presented in Extended Data Fig. 8.

### Western blotting

Quantitative western blots (Abby) with total protein were prepared and ran according to the manufacturer’s instructions using the manufacturer’s reagents. Cleaved caspase-3 antibody at 1:100 dilution (primary 9664S; CST) and undiluted anti-rabbit immunoglobulin G horeseradish peroxidase (ProteinSimple, cat. no. 042-406) were used. CRISPR-perturbed samples for cell death analysis were split on day 11 and harvested on day 12, both after transduction. Samples for western blotting by simple western blotting were collected as previously described, with the additional inclusion of the floating cell fraction^80^. As a control, we measured cleaved caspase-3 on induction of apoptosis with 1 µM staurosporine (CST, cat. no. 9953S) in SK-MEL-2 or 200 ng ml^−1^ of TNF (Immunotools, cat. no. 11343017) + 10 µM TPCA-1 (Selleckchem, cat. no. S2824) in BLM^83,84^.

### TIDE analysis

Genomic DNA was isolated from cell pellets using All-prep DNA/RNA kit (QIAGEN) according to the manufacturer’s instruction. A region around the sg*CDS2* no. 2 target site was amplified using NEBNext High Fidelity 2× PCR Master Mix (NEB) according to the manufacturer’s protocol. Primer sequences are available in Supplementary Table 7. PCR products were purified by Minelute (QIAGEN) according to the manufacturer’s protocol. Sanger sequencing (Macrogen) of each sample was performed in triplicate and chromatograms of each sg*CDS2* replicate were compared with each of three control sequences. Comparisons were made using the tracking of indels by decomposition (TIDE) website with settings of insertions and deletions (indels) size range 25 for NCI-H2030, LPS141 and K562 and 30 for SK-MEL-2 and MCF7 (ref. ^85^). The significance cut-off for calling indels was the default 0.001. The decomposition window was adjusted for each comparison. The frameshift percentage was calculated using the percentage without indels combined with significant indel percentages for multiples of three nucleotides.

### Animal studies

All animal studies were approved by the animal ethics committee of the NKI and performed under approved NKI CCD (Centrale Commissie Dierproeven) projects, according to the ethical and procedural guidelines established by the NKI and Dutch legislation (project no. AVD3010020172464 with protocol ID 9.1.10902 for the experiment in Fig. 2h and Extended Data Fig. 2f,g and project no. AVD30100202316772 with protocol ID 34.1.11655 for the experiment in Fig. 7c). Mice were housed in single-use standard cages at controlled filtered air humidity (55%), temperature (21 °C) and light cycle. All housing material, food and water were autoclaved or irradiated before use. Animal husbandry has been previously described^80^. Synthetic lethality in vivo was quantified like it was in vitro (described above) with the following adaptations: Puromycin selection was not continued in vivo*.* Two million melanoma cell mixes in 1:1 PBS Matrigel prepared on day 0 were injected subcutaneously into the flank of weight-randomized 13- to 15-week-old NOD-scid *Il2rγ*-null (Jax) mice. Mice were numbered and researchers were blinded from this step onwards. Tumor sizes were measured with calipers and the volume was calculated using length × width × width × 0.5. Tumors were harvested, digested and processed into homogeneous single-cell solutions. Then samples were stained for human B2M (BioLegend, cat. no. 316320) at 1:100 dilution to exclude mouse cells and analyzed by flow cytometry. Results were normalized to day 0 and sgControl. For each group one sample was unblinded during flow cytometry acquisition to set voltages and gates for all groups. Growth curves from one million subcutaneously injected unmixed melanoma cells were determined in the same manner as for the mixed cells.

### Genome-wide CRISPR perturbation

Screening was performed as published previously^82,86,87^. The one-vector Brunello library was amplified in liquid culture at 20,000× coverage and maintenance of the original sgRNA distribution was confirmed. Screen cells were transduced at a multiplicity of infection of 0.3–0.7 with polybrene (Sigma-Aldrich, cat. no. TR-1003-G) at 8 µg ml^−1^. Screens were performed at 200× coverage. Novaseq was used to sequence the screen samples and the amplified Brunello library. Sequencing primers with unique dual indices per sample were used (Supplementary Table 7).

To allow dropout of essential sgRNAs, cells were cultured for 10 d after Brunello library transduction. After this, single sgControl and sg*CDS2* lentivirus transduction cells were cultured for 18 d. The second-best sgRNA out of four guides was used to calculate the effect size. For SK-MEL-2, comparatively few cells were available as a result of lethality and no hits were detected. To ensure detection of any escape mechanism, a cryopreserved sample from day 7 was cultured until day 32 after single sgControl and sg*CDS2* transduction, and those results were used for the presented analyses.

The colony formation assays were started with screen cells on day 8. For K562, MCF7, NCI-H2030, LPS141 and SK-MEL-2 cell lines, 125,000, 125,000, 62,500, 31,250 and 125,000 cells were seeded, respectively. The Crystal Violet staining was done when control cells had reached confluence, which was after 4, 14, 12, 7 and 12 d, respectively. A seeding density corresponding to the longest culture time for MCF7 was chosen to ensure maximum sensitivity for detecting a potential difference. For K562 suspension cells, the cells were instead counted with Trypan Blue using an automatic cell counter (BioRad, cat. no. TC20). In addition, lethality was tracked from day 8 until day 19 using tracker cells and used to calculate rescue percentages. The quality control receiver operating characteristic area under the curve (ROC-AUC) was generated as described previously^86^. We used ROC-AUC because it is a standard metric for evaluating CRISPR screen performance, providing a clear measure of how well true positives are distinguished from background noise (true negatives).

### Lethality and synthetic lethality rescue experiments

For the synthetic lethality rescue experiments, the method to track synthetic lethality by tracking GFP positivity was used. The indicated cell mixes were generated 7 d after transduction with sgControl or sg*CDS2* lentivirus. After overnight attachment, treatment was started and samples were harvested to measure GFP positivity at the starting point. Cells were split and refreshed on day 3 and measured on day 7 after the start of treatment. Phospholipids were complexed with BSA. Stock solutions of 500 µM were generated by incubating phospholipid with 2.5% fatty-acid free BSA from Sigma-Aldrich (cat. no. A8806) in DMEM at 37 °C for 1 h. Liver phosphatidylcholine (PC; cat. no. 840055P-25MG) or PI (cat. no. 840042P-25MG) from Avanti Polar Lipids was used. Final GFP-negative to GFP-positive ratios were normalized to input ratios and sgControl ratios to obtain the synthetic lethal fraction. For rescue, the fold-change in the synthetic lethal fraction was calculated by dividing by the untreated synthetic lethal fractions and transformed into rescue percentages by subtraction from 1 and multiplication by 100. The resulting rescue percentages correspond to the part of the total synthetic lethality that was negated in the treated conditions. For the overnight lethality rescue, CRISPR-perturbed samples for cell death analysis were split on day 11 and harvested on day 12, both after transduction. PI was dissolved in medium and added to the cells at 2 mM.

### GSEAs

Hallmark GSEAs were performed using GSEA v.4.1.0 (Java tool) with the settings h.all.v2022.1.Hs.symbols.gmt, 25,000 permutations and No_Collapse on Pearson correlations generated as described from DepMap or TCGA data^88,89^. Gene set enrichment for the GO term processes was performed using the Stringdb ‘multiple proteins’ function on the list of 37 genes significant only in the 4 *CDS1*-negative cell lines^90^.

### Lipidomics

Quantitative targeted lipidomics was carried out using a flow-injection assay employing lipid class separation by differential mobility spectroscopy and selective multiple reaction monitoring (MRM) per lipid species (Lipidyzer platform). A detailed description of lipid extraction, software and the quantitative nature of the approach can be found elsewhere^91–93^. Briefly, after the addition of >60 deuterated internal standards, lipids were extracted using methyl *tert*-butyl ether. Organic extracts were subsequently dried under a gentle stream of nitrogen and reconstituted in running buffer. Lipids were subsequently analyzed using flow injection in MRM mode employing a Shimadzu Nexera series HPLC (high-performance liquid chromatograph) and a Sciex QTrap 5500 mass spectrometer. For further analysis, lipidomic data normalized to cell number were used to calculate the quadruplicate fold-changes for plotting and statistical analysis.

### Proteomics

A detailed sample- and data-processing protocol for generating the proteomic results is available in Supplementary Note. For plotting Fig. 5b and Extended Data Fig. 5a,c, log_2_-transformed protein group abundances were used that were available for each cell line in at least four out of four replicates in the sgControl or sg*CDS2* sample group. Differentially expressed proteins were determined using a two-sided Student’s *t*-test (thresholds: *P* < 0.05). For plotting Fig. 5c and Extended Data Fig. 5c, fold-changes relative to the average protein abundance per experiment were calculated for CDS1, CDS2 and calibration proteins. Then, for the CDS1 and CDS2 proteins, the fold-change to the median calibration protein levels per sample was calculated. For the CDS1 and CDS2 proteins, the fold-change to average abundance by experiment was recalculated and, respectively, replicates and experiments were averaged. With the combined data, the relative abundance of the CDS1 and CDS2 proteins to the highest included cancer cell line was calculated and plotted.

### Light microscopy

Live cells stained with BODIPY (2 µM, Thermo Fisher Scientific, cat. no. D3922) were imaged on a regularly maintained Zeiss Axio Observer Z1 microscope at the NKI imaging facility using phase contrast and fluorescence equipment. A home-built, temperature-controlled, live-cell chamber and 5% CO_2_ incubation system were used. Data were analyzed using custom ImageJ code. Briefly, for each condition, the low complexity area on phase contrast was excluded to calculate cell area and BODIPY fluorescence was used to calculate the lipid area. Cell lines were imaged 14 d (SK-MEL-2) or 20 d (NCI-H2030) after transduction (Fig. 6b). For imaging, cells were seeded several days in advance in a twofold dilution series of seven steps. At the time of imaging, different conditions were imaged at a similar density using the available dilution series.

### Electron microscopy

Correlative light electron microscopy (CLEM) was performed as described before^94,95^. Briefly, for CLEM 150- to 300-nm cryosections of cells were placed on to an EM grid, stained with Nile Red (Sigma-Aldrich, cat. no. 72485) and Hoechst 33342 (Thermo Fisher Scientific, cat. no. H3570) and imaged with a Wide-field Fluorescence Microscope (Leica DM6, ×100 oil objective). After washing and contrasting for EM purposes, the identical grid was imaged using a transmission electron microscope (Thermo Fisher Scientific, Talos, cat. no. L120c with a Ceta 16M camera).

### Statistics and replication

Significance was tested and depicted as indicated. Two-sided tests were employed. To ensure reproducibility, we addressed experimental variation by including multiple replicates and biological variation by including multiple cell lines and we employed multiple methods whenever possible. Replicates were generated during the same period in time and independent experiments were performed during different moments in time. For admixing, experimental replicates were cultured independently from the start to the finish. The assumption of normality was kept at a minimum by using nonparametric tests for large datasets (Kruskal–Wallis test and Mann–Whitney *U* test). Data processing was always verified by a second researcher. Complex bioinformatic analyses were redone independently.

### Reporting summary

Further information on research design is available in the Nature Portfolio Reporting Summary linked to this article.

## Online content

Any methods, additional references, Nature Portfolio reporting summaries, source data, extended data, supplementary information, acknowledgements, peer review information; details of author contributions and competing interests; and statements of data and code availability are available at 10.1038/s41588-025-02221-2.

## Supplementary information

Supplementary Information Supplementary Note A detailed sample- and data-processing protocol for generating the proteomic results.

Reporting Summary

Supplementary Tables Supplementary Table 1 Synthetic lethality scores and cancer-specificity scores for the top synthetic lethal gene pairs. Supplementary Table 2 The CRISPR screen data. Supplementary Table 3 The proteomic data. Supplementary Table 4 The lipidomic data. Supplementary Table 5 STR profiles generated from the cell lines used in the present study. Supplementary Table 6 Methodological details for SEER lineage matching. Supplementary Table 7 Primer, sgRNA and other sequences used in the present study.

## Source data

Source Data Fig. 1 Plotted data and statistics: unprocessed data and code are available on repositories.

Source Data Fig. 2 Plotted data and statistics: unprocessed data and code are available on repositories.

Source Data Fig. 3 Plotted data and statistics: unprocessed data and code are available on repositories.

Source Data Fig. 4 Plotted data and statistics: unprocessed data and code are available on repositories.

Source Data Fig. 5 Plotted data and statistics: unprocessed data and code are available on repositories.

Source Data Fig. 6 Plotted data and statistics: unprocessed data and code are available on repositories.

Source Data Fig. 7 Plotted data and statistics: unprocessed data and code are available on repositories.

Source Data Extended Data Fig. 1 Plotted data and statistics: unprocessed data and code are available on repositories.

Source Data Extended Data Fig. 2 Plotted data and statistics: unprocessed data and code are available on repositories.

Source Data Extended Data Fig. 3 Plotted data and statistics: unprocessed data and code are available on repositories.

Source Data Extended Data Fig. 4 Plotted data and statistics: unprocessed data and code are available on repositories.

Source Data Extended Data Fig. 5 Plotted data and statistics: unprocessed data and code are available on repositories.

Source Data Extended Data Fig. 6 Plotted data and statistics: unprocessed data and code are available on repositories.

Source Data Extended Data Fig. 7 Plotted data and statistics: unprocessed data and code are available on repositories.

## Data Availability

Tables presenting a selection of plotted source data, unplotted source data and methodological data are available (Supplementary Tables 1–7). To allow large file sizes, unprocessed source data are available on repositories. Unprocessed proteomic data are available on our entry in the Proteomics IDEntifications repository, under accession no. PXD045833 (ref. ^96^). All other unprocessed source data for the present study are available via figshare at 10.6084/m9.figshare.27951504 (ref. ^97^). Detailed instructions for downloading the specific public data files used in our software notebooks, including links to repositories when possible, are available via Zenodo at 10.5281/zenodo.15194712 (ref. ^98^). Briefly here, we used the HGNC database (https://www.genenames.org); the UniProt database (https://www.uniprot.org); ENCODE data via Harmonizone (https://maayanlab.cloud/harmonizone)^51^; DepMap data from 21Q2 and 23Q4 on figshare and the DepMap portal (https://depmap.org/portal)^33,34,37,38,75^; reprocessed TCGA RNA-seq data from Gene Expression Omnibus, accession no. GSE62944 (ref. ^76^); original RNA-seq and clinical CDR TCGA data^35^ from the Genomic Data Commons website (https://gdc.cancer.gov; downloaded 12 January 2023); bulk expression gene TPM data from the GTEx^36^ portal (https://gtexportal.org/home) (v.8); Satpathy et al. lung proteome data and tumor normal mapping from Proteomic Data Commons, accession no. PCD000234 (ref. ^47^); untreated (0 h) BLM and SK-MEL-147 cell-line RNA-seq data from refine.bio, accession no. SRP132830 (ref. ^82^); and 2013–2017 US SEER cancer mortality data from the SEER website (https://seer.cancer.gov; downloaded 18 January 2023; copy in Supplementary Table 6). Source data are provided with this paper.

## Code availability

For the computational analyses for which custom code was used, software notebooks designed to ease reproduction are available via Zenodo at 10.5281/zenodo.15194712 (ref. ^98^). Either files required to run the provided software are directly supplied or detailed instructions for downloading are supplied on our Zenodo entry.
