# Supplementary material for: Cytidine diphosphate diacylglycerol synthase 2 is a synthetic lethal target in mesenchymal-like cancers
Source: Nat Genet. 2025 Jul 4;57(7):1659–71. doi: 10.1038/s41588-025-02221-2 (PMC12283369; doi:10.1038/s41588-025-02221-2)
Supplement: Supplementary file 1 — Supplementary Note A detailed sample- and data-processing protocol for generating the proteomic results. [file 41588_2025_2221_MOESM1_ESM.pdf]

nature genetics

Supplementary information https://doi.org/10.1038/s41588-025-02221-2

Cytidine diphosphate diacylglycerol synthase 2 is a synthetic lethal target in mesenchymal-like cancers

In the format provided by the authors and unedited

Proteomics sample processing protocol

Frozen cell pellets were heated for 7 min. at 95^{0}C in 1x S-Trap Lysis buffer (5% SDS, 50 mM TEAB pH 8.5), after which DNA was sheared by probe sonication. Aliquots comprising 50 µg of protein were reduced with 20 mM DTT (20 min. at 55^{0}C) and alkylated with 40 mM iodoacetamide (30 min. at room temperature in the dark), after which proteins were digested overnight with trypsin (Sigma-Aldrich; enzyme/substrate ratio 1:10) on S-Trap Micro spin columns according to the manufacturer's instructions (ProtiFi, NY, USA). Peptides were eluted, vacuum-dried and stored at -80°C until LC-MS/MS analysis. For **Fig. 5b**, **Extended Data Fig. 5a, c**: LC-MS/MS was performed by nanoLC-MS/MS on an Orbitrap Exploris 480 mass spectrometer (Thermo Scientific) connected to a Proxeon nLC1200 system. Peptides were directly loaded onto the analytical column (ReproSil-Pur 120 C18-AQ, 2.4µm, 75 µm × 500 mm, packed in-house) and eluted at 250 nL/min in a 90-minutes gradient containing a non-linear increase from 6% to 30% solvent B (solvent A was 0.1% formic acid/water and solvent B was 0.1% formic acid/80% acetonitrile). The Exploris 480 was run in data-independent acquisition (DIA) mode, with full MS resolution set to 120,000 at m/z 200, MS1 mass range was set from 350-1400, normalized AGC target was 300% and maximum IT was 45ms. DIA was performed on precursors from 400-1000 in 48 windows of 13.5 Da with an overlap of 1 Da. Resolution was set to 30,000 and normalized CE was 27. For **Fig. 5c, Extended Data Fig. 5c, d**: nanoLC-MS/MS was performed on an Orbitrap Astral mass spectrometer (Thermo Scientific) connected to a Vanquish Neo nano-LC system (Thermo Scientific). The Vanquish Neo was operated in the trap-and-elute mode and peptides were loaded onto a Pepmap 100 C18 5µm trap column (300µm x 5mm, Thermo Scientific), before separation on the analytical column (AUR3-25075C18-TS, 1.7µm/75µm x 25cm, IonOpticks AU) mounted into an Easyspray ion source (Thermo Scientific). Peptides were eluted at a flow rate of 0.4 µL/min in a 36-min effective gradient, containing a non-linear increase from 8% to 45% solvent B, followed by a 0.4-min ramp to 99% solvent B and 5.4-min wash at 0.5 µL/min flow rate at the end. The column was equilibrated using the "fast equilibration" script in combined control mode with a 1450 bar pressure limit. The mass spectrometer was run in data-independent acquisition (DIA) mode, with full MS scans being collected in the Orbitrap analyzer with 240,000 resolution at m/z 200 over a 380-980 m/z range. Default charge state was 2+, the normalized AGC target was set to 500% (equivalent to 5e6 charges) and the maximum injection time was 5 ms. For DIA MS2, a normalized HCD collision energy of 25% was applied to a 380-980 m/z precursor range using non-overlapping isolation windows of 2Th, with window placement optimization turned on. Scans were acquired in the Astral analyzer over a 100-1000 m/z range, with the normalized AGC target set to 500% (equivalent to 5e4 charges) and a maximum injection time of 3 ms.

Proteomics data processing protocol

For **Fig. 5b**, **Extended Data Fig. 5a, c**: RAW files were analyzed with DIA-NN (version 1.8) using standard settings. A spectral library was generated from the Swissprot human database (version 2022_02; 20,375 entries) by selecting 'FASTA digest for library-free search'; Trypsin/P was specified as protease specificity allowing a maximum of 1 miscleavage; N-terminal excision (M) and carbamidomethylation (C) were selected as variable and fixed modifications, respectively, and "match between runs" was applied. Protein group abundances were extracted from the DIA-NN result files, imported into Perseus (1.6.15.0) and Log2-transformed. For each cell line, values were filtered for presence in at least 4 out of 4 replicates in at least the sgControl or sg*CDS2* sample group. Missing values were replaced by an imputation-based normal distribution using a width of 0.3 and a downshift of 2.4. Differentially expressed proteins were determined using a two-sided t-test (thresholds: p-value<0.05). For **Fig. 5c, Extended Data Fig. 5c, d**: RAW files were analyzed with DIA-NN (version 1.9.1) or Proteome Discoverer (PD) (Thermo Scientific, version 3.1.0.638) using Chimerys on Ardia (version 1.0.0-qf.1.) using the same settings as described above, with a few modifications. A spectral library was generated from the Swissprot human database containing reviewed canonical and isoform sequences (release 2023_10; 42,359 entries). The maximum number of allowed protease miscleavages was 1 (DIA-NN) or 3 (PD) and in all cases oxidation (M) and carbamidomethylation (C) were selected as variable and fixed modifications, respectively. In the Fragment Ions Quantifier node of PD, the normalization mode was set to 'total peptide amount' and summed abundances were used for protein abundance calculation.
