# Supplementary material for: Cytidine diphosphate diacylglycerol synthase 2 is a synthetic lethal target in mesenchymal-like cancers
Source: Nat Genet. 2025 Jul 4;57(7):1659–71. doi: 10.1038/s41588-025-02221-2 (PMC12283369; doi:10.1038/s41588-025-02221-2)
Supplement: Supplementary file 2 — Reporting Summary [file 41588_2025_2221_MOESM2_ESM.pdf]

nature portfolio

Corresponding author(s): Peeper D.S.

Last updated by author(s): Apr 15, 2025

Reporting Summary

Nature Portfolio wishes to improve the reproducibility of the work that we publish. This form provides structure for consistency and transparency in reporting. For further information on Nature Portfolio policies, see our Editorial Policies and the Editorial Policy Checklist.

Statistics

For all statistical analyses, confirm that the following items are present in the figure legend, table legend, main text, or Methods section.

n/a	Confirmed	
☐	☒	The exact sample size (*n*) for each experimental group/condition, given as a discrete number and unit of measurement
☐	☒	A statement on whether measurements were taken from distinct samples or whether the same sample was measured repeatedly
☐	☒	The statistical test(s) used AND whether they are one- or two-sided<br>*Only common tests should be described solely by name; describe more complex techniques in the Methods section.*
☐	☒	A description of all covariates tested
☐	☒	A description of any assumptions or corrections, such as tests of normality and adjustment for multiple comparisons
☐	☒	A full description of the statistical parameters including central tendency (e.g. means) or other basic estimates (e.g. regression coefficient) AND variation (e.g. standard deviation) or associated estimates of uncertainty (e.g. confidence intervals)
☐	☒	For null hypothesis testing, the test statistic (e.g. *F*, *t*, *r*) with confidence intervals, effect sizes, degrees of freedom and *P* value noted<br>*Give P values as exact values whenever suitable.*
☒	☐	For Bayesian analysis, information on the choice of priors and Markov chain Monte Carlo settings
☒	☐	For hierarchical and complex designs, identification of the appropriate level for tests and full reporting of outcomes
☐	☒	Estimates of effect sizes (e.g. Cohen's *d*, Pearson's *r*), indicating how they were calculated

Our web collection on statistics for biologists contains articles on many of the points above.

Software and code

Policy information about availability of computer code

Data collection	Flow cytometry: FACSDiva (v8.0), Live cell imaging: ZEN (3.5), Quantitative western: Compass for SW (6.1.0), Proteomics: DIA-NN (version 1.8 or 1.9.1), Proteome Discoverer (Thermo Scientific, version 3.1.0.638) using Chimerys on Ardia (version 1.0.0-qf.1.), Lipidomics: Shotgun Lipidomics Assistant SLA (1.3), Genome-wide screen sequencing: BCLConvert (v3.9.2-3.e17), Perl (5.010), xcalibr (0.12031), Combined light and electron microscopy: LAS X (3.7.3.23245), MAPS (3.17), qPCR: Quantstudio (1.5.2)
Data analysis	Flow cytometry: Flowjo (v10.6.0), General data analysis: Excel (16.71), R (4.2.2), RStudio (2023.03.0+386), GraphPad Prism (9.4.1 or 10.0.3), Genome wide screen analysis: MAGeCK v0.5.9.5, Live cell imaging analysis: FIJI 2.14.0, Java 1.8.0, ImageJ 1.54f, Gene set enrichment analysis: GSEA (4.1.0), Proteomics analysis: Perseus (1.6.15.0), Combined light and electron microscopy analysis: ICY ec-CLEM-v2 (ICY-82440), Protein visualization: ChimeraX version 1.5rc202210210730<br><br>Quantitative elements of figures were generated using GraphPad Prism or Flowjo. Visual elements of graphs and plots were designed and generated in Adobe Illustrator (26.2). Details can be found in the methods section.<br><br>Code availability<br>For the computational analyses for which custom code was used software notebooks designed to ease reproduction are available via Zenodo at https://doi.org/10.5281/zenodo.15194712. Files required to run the provided software are either directly supplied or detailed instructions for downloading are supplied on our Zenodo entry.

For manuscripts utilizing custom algorithms or software that are central to the research but not yet described in published literature, software must be made available to editors and reviewers. We strongly encourage code deposition in a community repository (e.g. GitHub). See the Nature Portfolio guidelines for submitting code & software for further information.

Data

Policy information about availability of data

All manuscripts must include a data availability statement. This statement should provide the following information, where applicable:

- Accession codes, unique identifiers, or web links for publicly available datasets
- A description of any restrictions on data availability
- For clinical datasets or third party data, please ensure that the statement adheres to our policy

Data availability
Plotted data and statistics are available as source data here. Tables presenting a selection of plotted source data, unplotted source data and methodological data are available here (Supplementary Tables 1-7). To allow large file sizes, unprocessed source data are available on repositories. Unprocessed proteomic data are available on our entry in the Proteomics IDEntifications repository under accession PXD045833 (Ref. 1). All other unprocessed source data for this study are available on our Figshare entry at https://doi.org/10.6084/m9.figshare.27951504 (Ref. 2). Our Zenodo entry at https://doi.org/10.5281/zenodo.15194712 (Ref. 3) also contains detailed instructions for downloading the specific public data files used in our software notebooks, including links to repositories when possible. Briefly here, we used the HGNC database; the Uniprot database; ENCODE data via Harmonizome (Ref. 4); DepMap data from 21Q2, 23Q4 on Figshare and the DepMap portal (Refs. 5-9); reprocessed TCGA RNA sequencing data from Gene Expression Omnibus accession GSE62944 (Ref. 10); original RNA sequencing and clinical CDR TCGA data from the Genomic Data Commons website (downloaded January 12th 2023, Ref. 11); bulk expression gene TPM data from the GTEx portal (V8, Ref. 12); Satpathy et al. lung proteome data and tumor normal mapping from Proteomic Data Commons accession PCD000234 (Ref. 13); untreated (0 hour) BLM and SK-MEL-147 cell line RNA sequencing data from refine.bio accession SRP132830 (Ref. 14); and 2013-2017 United States SEER cancer mortality data from the SEER website (downloaded January 18th 2023, copy in Supplementary Table 6).

1. Arnoldus, T. et al. CDS2 is a synthetic lethal target in mesenchymal-like cancers (proteomic dataset). PRIDE https://www.ebi.ac.uk/pride/archive/projects/PXD045833 (2025).
2. Arnoldus, T. et al. CDS2 is a synthetic lethal target in mesenchymal-like cancers (dataset). Figshare https://doi.org/10.6084/m9.figshare.27951504 (2025).
3. Arnoldus, T. et al. CDS2 is a synthetic lethal target in mesenchymal-like cancers (software notebooks). Zenodo https://doi.org/10.5281/zenodo.15194712 (2025).
4. Feingold, E. A. et al. The ENCODE (ENCyclopedia of DNA Elements) Project. Science 306, 636–640 (2004).
5. Meyers, R. M. et al. Computational correction of copy number effect improves specificity of CRISPR-Cas9 essentiality screens in cancer cells. Nat. Genet. 49, 1779–1784 (2017).
6. DepMap 21Q2 Public. Figshare https://figshare.com/articles/dataset/DepMap_21Q2_Public/14541774/2 (2021).
7. Nusinow, D. P. et al. Quantitative proteomics of the Cancer Cell Line Encyclopedia. Cell 180, 387–402.e16 (2020).
8. Ghandi, M. et al. Next-generation characterization of the Cancer Cell Line Encyclopedia. Nature 569, 503–508 (2019).
9. DepMap 23Q4 Public. Figshare https://plus.figshare.com/articles/dataset/DepMap_23Q4_Public/24667905 (2023).
10. Rahman, M. et al. Alternative preprocessing of RNA-Sequencing data in the Cancer Genome Atlas leads to improved analysis results. Bioinformatics 31, 3666–3672 (2015).
11. Weinstein, J. N. et al. The cancer genome atlas pan-cancer analysis project. Nat. Genet. 45, 1113–1120 (2013).
12. Lonsdale, J. et al. The Genotype-Tissue Expression (GTEx) project. Nat. Genet. 45, 580–585 (2013).
13. Satpathy, S. et al. A proteogenomic portrait of lung squamous cell carcinoma. Cell 184, 4348–4371.e40 (2021).
14. Vredevoogd, D. W. et al. Augmenting immunotherapy impact by lowering tumor TNF cytotoxicity threshold. Cell 178, 585–599.e15 (2019).

Research involving human participants, their data, or biological material

Policy information about studies with human participants or human data. See also policy information about sex, gender (identity/presentation), and sexual orientation and race, ethnicity and racism.

Reporting on sex and gender	not applicable
Reporting on race, ethnicity, or other socially relevant groupings	not applicable
Population characteristics	not applicable
Recruitment	not applicable
Ethics oversight	not applicable

Note that full information on the approval of the study protocol must also be provided in the manuscript.

Field-specific reporting

Please select the one below that is the best fit for your research. If you are not sure, read the appropriate sections before making your selection.

☒ Life sciences ☐ Behavioural & social sciences ☐ Ecological, evolutionary & environmental sciences

For a reference copy of the document with all sections, see nature.com/documents/nr-reporting-summary-flat.pdf

Life sciences study design

All studies must disclose on these points even when the disclosure is negative.

Sample size	The sample size for the in vivo experiments was determined upfront using power calculation to comply with ethical guidelines.<br><br>For the admixing experiment:<br>At an effect size of 80% (in vitro this was 85% & 98%) 5 mice per group should yield 95% power to detect our difference at p = 0.05 with a standard deviation of up to 35 percentage points (in vitro this was 0.3-2%). The power calculation assumes data is collected for all mice from all groups. An additional mice per group was included to ensure sufficient power even if data for a mice in one of the groups was unavailable for any reason resulting in 6 mice per group for all groups.<br>For the unmixed experiment:<br>Using the growth curve data from the admixing experiment our power calculation yielded 7 mice per group. An additional mice per group was included to ensure sufficient power even if data for a mice in one of the groups was unavailable for any reason. Furthermore because now male and female mice were included an additional mouse per group was added to compensate for any potential variation from this added variable.<br><br>For all other experiments whether the sample size was sufficient to detect the effect size above background became apparent when performing a statistical test on the results. Such tests require at least 3 replicates and generally standard triplicates or quadruplicates were used in terms of replicates.
Data exclusions	None.
Replication	To ensure reproducibility, we addressed experimental variation by including multiple replicates, we addressed biological variation by including multiple cell lines and we employed multiple methods whenever possible. Replicates were generated during the same period in time and independent experiments were performed during different moments in time. Data processing was always verified by a second researcher. Complex bioinformatic analyses were redone independently. Details are in the manuscript. Specifically, we list the number of replicates, independent experiments, cell lines and other relevant details in Figure legends by panel. Furthermore, we explain when multiple techniques or observations supported the same conclusion in the text.
Randomization	The mice for in vivo experiments were randomized into the groups by weight. For other experiments randomization was not applicable. For example, in standard cell culture millions of cells from a cancer cell line can be divided from a single homogeneous aqueous solution over the different conditions. Such a solution can be mixed by flipping the tube or pipetting.
Blinding	Researchers were blinded after injection of mice for the in vivo experiments. For in vitro experiments this is not applicable. For those experiments we used well-controlled objective quantitative methods to exclude bias.

Reporting for specific materials, systems and methods

We require information from authors about some types of materials, experimental systems and methods used in many studies. Here, indicate whether each material, system or method listed is relevant to your study. If you are not sure if a list item applies to your research, read the appropriate section before selecting a response.

Materials & experimental systems

n/a	Involved in the study	
☐	☒	Antibodies
☐	☒	Eukaryotic cell lines
☒	☐	Palaeontology and archaeology
☐	☒	Animals and other organisms
☒	☐	Clinical data
☒	☐	Dual use research of concern
☒	☐	Plants

Methods

n/a	Involved in the study	
☒	☐	ChIP-seq
☐	☒	Flow cytometry
☒	☐	MRI-based neuroimaging

Antibodies

Antibodies used	Cleaved caspase-3 (9664S, CST) used 1:100, Anti-Rabbit IgG HRP (042-406, ProteinSimple) used undiluted, human B2M (316320, Biolegend) used 1:100
Validation	Cleaved caspase-3 (9664S, CST), Anti-Rabbit IgG HRP (042-406, ProteinSimple)<br><br>As a positive control, we analyzed cleaved caspase-3 levels upon induction of apoptosis by TPCA-1+TNF (BLM) or staurosporine (SK-MEL-2). Use of this cleaved caspase-3 antibody was previously established in the Peeper lab [1, 2] and numerous other labs (5550 citations listed on https://www.cellsignal.com/products/primary-antibodies/cleaved-caspase-3-asp175-5a1e-rabbit-mab/9664). Use of Anti-Rabbit IgG was previously established by ProteinSimple with their equipment (DM-001, ProteinSimple).<br><br>Human B2M (316320, Biolegend)

Human B2M antibody was tested on material from an in vivo experiment in NOD-Scid IL2Rgnull (Jax) mice. Material from human cell line tumor, adjacent healthy mouse tissue and mixes of both were utilized to validate its use to separate human from mouse cells. It was also tested on mouse and human cell lines to confirm its specificity for human cells. It was also tested on BLM and SK-MEL-147 prior to the in vivo experiment to verify their positivity. Numerous citations on the clone 2M2 conjugated to various fluorochromes are listed on https://www.biolegend.com/fr-fr/search-results?Clone=2M2.

1. Vredevoogd, D. W. et al. Augmenting Immunotherapy Impact by Lowering Tumor TNF Cytotoxicity Threshold. Cell 178, 585-599.e15 (2019).
2. Lin, C., Traets, J. J. H., Vredevoogd, D. W., Visser, N. L. & Peeper, D. S. TSC2 regulates tumor susceptibility to TRAIL-mediated T-cell killing by orchestrating mTOR signaling. EMBO J. 42, e111614 (2023).

Eukaryotic cell lines

Policy information about cell lines and Sex and Gender in Research

Cell line source(s)

MCF7, A431, NCI-H1975, Jurkat, K562, NCI-H2030, SK-MEL-2, LPS141, D10, BLM, A375, SK-MEL-147, A549, CAKI1, COLO679: all from Peeper lab stocks.

Authentication

Cell lines were authenticated using the STR profiling kit from Promega (B9510). BLM, CAKI1, COLO679 and A549 were profiled by Eurofins. Public references were available for all cell lines except SK-MEL-147 and BLM. For SK-MEL-147 a reference was requested via private correspondence [1]. Our STR results are included with the manuscript.

1. Hanniford, D. et al. Epigenetic Silencing of CDR1as Drives IGF2BP3-Mediated Melanoma Invasion and Metastasis. Cancer Cell 37, 55-70.e15 (2020).

Mycoplasma contamination

We used mycoplasma negative cells by screening mycoplasma monthly [1].
1. Young, L., Sung, J. & Masters, J. R. Detection of mycoplasma in cell cultures. Nat. Protoc. 5, 929–934 (2010).

Commonly misidentified lines
(See ICLAC register)

None of the included cell lines are in the ICLAC register.

Animals and other research organisms

Policy information about studies involving animals; ARRIVE guidelines recommended for reporting animal research, and Sex and Gender in Research

Laboratory animals

NOD-scid Il2rγ-null (Jax) mice were used.

Wild animals

No wild animals were used.

Reporting on sex

Sex was not considered as a factor in any experiment or analysis.

Field-collected samples

No field-collected samples were used.

Ethics oversight

All animal studies were approved by the animal ethics committee of the Netherlands Cancer Institute (NKI) and performed under approved NKI CCD (Centrale Commissie Dierproeven) projects according to the ethical and procedural guidelines established by the NKI and Dutch legislation.

Note that full information on the approval of the study protocol must also be provided in the manuscript.

Plants

Seed stocks

Report on the source of all seed stocks or other plant material used. If applicable, state the seed stock centre and catalogue number. If plant specimens were collected from the field, describe the collection location, date and sampling procedures.

Novel plant genotypes

Describe the methods by which all novel plant genotypes were produced. This includes those generated by transgenic approaches, gene editing, chemical/radiation-based mutagenesis and hybridization. For transgenic lines, describe the transformation method, the number of independent lines analyzed and the generation upon which experiments were performed. For gene-edited lines, describe the editor used, the endogenous sequence targeted for editing, the targeting guide RNA sequence (if applicable) and how the editor was applied.

Authentication

Describe any authentication procedures for each seed stock used or novel genotype generated. Describe any experiments used to assess the effect of a mutation and, where applicable, how potential secondary effects (e.g. second site T-DNA insertions, mosiacism, off-target gene editing) were examined.

Flow Cytometry

Plots

Confirm that:

☒ The axis labels state the marker and fluorochrome used (e.g. CD4-FITC).

☒ The axis scales are clearly visible. Include numbers along axes only for bottom left plot of group (a 'group' is an analysis of identical markers).

☒ All plots are contour plots with outliers or pseudocolor plots.

☒ A numerical value for number of cells or percentage (with statistics) is provided.

Methodology

Sample preparation	Generally, for admixing experiments cancer cell lines cultured in 12-well plates were harvested and around 100,000 cells were placed in v-bottom 96 well plates with gaps between wells (781601, BRAND). For the in vivo experiments tumors were cut into small pieces, digested and around 100,000 cells were placed in v-bottom 96 well plates (781601, BRAND).<br><br>One of several live/dead dyes was always included: DAPI (D9542-50MG, Sigma), PI (537059-100MG, Merck), DRAQ7 (D15106, Thermofisher) and fixable near IR (L34976, Thermofisher). 0.1% BSA in PBS was used as a washing and staining solution. Samples were washed three times (including resuspension as one wash) in 0.1% BSA in PBS, once for dead cell experiments to preserve dead cells and six times for in vivo samples to reduce post-digestion debris. Antibody staining was performed for 30 minutes at 4 degrees Celsius in 100 µL staining solution with 1 µL of antibody solution. Samples were washed three times after antibody staining to remove excess antibody.
Instrument	One of the following:<br><br>LSRII SORP (BD Biosciences)<br>The LSRII is a Special Order Research Product (SORP) from BD Biosciences. This analytical flow cytometer is equipped with 5 lasers and can detect up to 18 fluorescent labels from a sample.<br><br>Fortessa 1 (BD Biosciences) / Fortessa 2 (BD Biosciences)<br>Fortessa 1/2 is equipped with 4 lasers, and can be used to detect up to 16 fluorescent labels per sample.<br><br>These function the same for our purposes. The extra laser of the LSRII SORP was not utilized.
Software	Collection using FACSDiva (v8.0). Analysis using Flowjo (v10.6.0), Excel (16.71) and Graphpad Prism (9.4.1).
Cell population abundance	Flow cytometry was used to track relative abundance of admixed cell populations over time. Generally, cells were admixed close to 50/50 at start of experiment and 10,000 events in live cell gates were collected to track relative abundance. Flow cytometry was also used to measure the fraction of dead cells in a sample. Dead cell populations were abundant in the conditions of interest (percentages presented in the manuscript).
Gating strategy	The actual gating strategies are best shown visually, and are therefore available in Extended Data Figure 8. Generally we applied standard FSC versus SSC cell gate to exclude debris, followed by a SSC-H versus SSC-A single cell gate to exclude cell clumps, followed by a FSC versus live-dead dye gate to exclude dead cells and then gating for populations of interest. For detecting dead cells the gating was adapted. We applied a large FSC versus SSC cell gate to exclude only very large clumps (keeping dead cells), followed by a long SSC-H versus SSC-A single cell gate to exclude cell clumps (keeping dead cells again) and then the gating for populations of interest.

☒ Tick this box to confirm that a figure exemplifying the gating strategy is provided in the Supplementary Information.
